# A phylogenetically distinct lineage of *Pyrenopeziza brassicae* associated with chlorotic leaf spot of Brassicaceae in North America

**DOI:** 10.1111/ppa.13137

**Published:** 2020-01-22

**Authors:** Shannon M. Carmody, Kevin M. King, Cynthia M. Ocamb, Bart A. Fraaije, Jon S. West, Lindsey J. du Toit

**Affiliations:** ^1^ Department of Plant Pathology Washington State University Mount Vernon WA USA; ^2^ Rothamsted Research Harpenden UK; ^3^ Department of Botany and Plant Pathology Oregon State University Corvallis OR USA

**Keywords:** Brassicaceae, chlorotic leaf spot, light leaf spot, Pacific Northwest USA, phylogenetic lineage, *Pyrenopeziza brassicae*

## Abstract

Light leaf spot, caused by the ascomycete *Pyrenopeziza brassicae*, is an established disease of Brassicaceae in the United Kingdom (UK), continental Europe, and Oceania (OC, including New Zealand and Australia). The disease was reported in North America (NA) for the first time in 2014 on *Brassica* spp. in the Willamette Valley of western Oregon, followed by detection in *Brassica juncea* cover crops and on *Brassica rapa* weeds in northwestern Washington in 2016. Preliminary DNA sequence data and field observations suggest that isolates of the pathogen present in NA might be distinct from those in the UK, continental Europe, and OC. Comparisons of isolates from these regions using genetic (multilocus sequence analysis, *MAT* gene sequences, and rep‐PCR DNA fingerprinting), pathogenic (*B. rapa* inoculation studies), biological (sexual compatibility), and morphological (colony and conidial morphology) analyses demonstrated two genetically distinct evolutionary lineages. Lineage 1 comprised isolates from the UK, continental Europe, and OC, and included the *P. brassicae* type specimen. Lineage 2 contained the NA isolates associated with recent disease outbreaks in the Pacific Northwest region of the USA. Symptoms caused by isolates of the two lineages on *B. rapa* and *B. juncea* differed, and therefore “chlorotic leaf spot” is proposed for the disease caused by Lineage 2 isolates of *P. brassicae*. Isolates of the two lineages differed in genetic diversity as well as sensitivity to the fungicides carbendazim and prothioconazole.

## Graphical abstract

*Pyrenopeziza brassicae* was first found on *Brassica* spp. in North America (NA) in 2014. The consolidated species concept showed NA isolates are in a distinct evolutionary lineage compared to isolates from continental Europe and the UK.

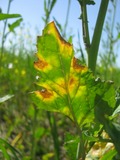

## 1 INTRODUCTION

Light leaf spot, caused by the ascomycete *Pyrenopeziza brassicae* (anamorph *Cylindrosporium concentricum*), is an economically important disease of many Brassicaceae (Rawlinson *et al.*, 1978; Centre for Agriculture and Biosciences International [CABI], 2015). The pathogen is widespread geographically, having been reported in Asia (Japan and the Philippines), continental Europe (including France, Germany, and Poland), the United Kingdom (UK), and Oceania (OC, including Australia and New Zealand). Light leaf spot is one of the most important diseases of *Brassica napus* (oilseed rape) in the UK and northern parts of Europe (Boys *et al.*, 2007). However, excluding a single unconfirmed record from Oregon State in 1998 (Phytosanitary Alert System, 2015), light leaf spot had not previously been documented in North America (NA). The disease was first found on *Brassica juncea*, *B. napus*, and *Brassica rapa* in six counties in the Willamette Valley of western Oregon in 2014 (Ocamb *et al.*, 2015), and subsequently has been detected in additional counties on multiple Brassicaceae genera and species in western Oregon (Claassen, 2016). In 2016, light leaf spot was detected in *B. juncea* cover crops and on *B. rapa* weeds (birdsrape mustard) in three counties in northwestern Washington (Carmody *et al.*, 2016). Isolates of *P. brassicae* obtained from diverse Brassicaceae genera and species in Oregon and Washington were confirmed to be pathogenic on *B. juncea*, *B. napus*, *B. oleracea*, and *B. rapa* (Claassen, 2016; Carmody, 2017). Light leaf spot can cause reduced photosynthesis, stunting, pod shatter (for seed crops), and associated declines in yield (Claassen, 2016; Karandeni Dewage *et al.*, 2018). Thus, the relatively recent appearance of light leaf spot in Oregon and Washington could pose a threat to production of economically important crops of the many diverse types of Brassicaceae grown in the Pacific Northwest USA, including *B. napus*, *B. oleracea*, and *B. rapa* crops (Inglis *et al.*, 2013; Phytosanitary Alert System, 2015).

Light leaf spot appears to have undergone very recent, rapid, and invasive spread in the US Pacific Northwest given that: (a) the disease was not observed in surveys of *Brassica* and *Raphanus* crops in Oregon from 2010 to 2013 (Ocamb, 2014); (b) light leaf spot was first reported in Oregon in 2014 (Ocamb *et al.*, 2015) and is now widespread across parts of western Oregon (Claassen, 2016); and (c) the disease was found in three counties in northwestern Washington in 2016 (Carmody, 2017). The origins of the isolates associated with these recent outbreaks in NA are not yet known. As is the case with many newly emerging plant diseases, the outbreaks in NA might have resulted from introduction of the pathogen (Anderson *et al.*, 2004) into the Pacific Northwest US, perhaps via infected planting material, given evidence for the seedborne and seed transmitted nature of the fungus (Carmody, 2017; Carmody and du Toit, 2017). If the pathogen was introduced recently to NA, candidate regions of origin of the pathogen include areas where the disease has long been reported, such as the UK, continental Europe, and OC (Rawlinson *et al.*, 1978; CABI, 2015). However, a preliminary comparison of sequences of the internal transcribed spacer (ITS) region of ribosomal DNA (rDNA) of five NA isolates suggested that they were distinct genetically from European and UK isolates, as the sequences only had 95% nucleotide similarity (Carmody, 2017). The *β‐tubulin* gene sequences of the same NA isolates had 98% nucleotide similarity to isolates of *P. brassicae* from the UK and continental Europe (Carmody, 2017). This initial evidence that the light leaf spot pathogen isolates in NA might be distinct genetically from those from continental Europe and the UK highlighted the need to assess the pathogen on a larger temporal and spatial scale.

Dispersal of *P. brassicae* inoculum during the growing season in areas where this pathogen is established is considered mainly to be by short distance splash‐dispersal of asexual conidia, with multiple (polycyclic) rounds of host infection (Gilles *et al.*, 2001; Karandeni Dewage *et al.*, 2018). In addition, wind‐dispersed ascospores are released into the air forcibly from apothecia that form on infected host debris, typically in late summer and autumn (Cheah *et al.*, 1982; Gilles *et al.*, 2001). Ascospores are thought to act as primary sources of inoculum that initiate light leaf spot outbreaks in the UK and continental Europe (Karolewski *et al.*, 2012). Sexual reproduction by *P. brassicae* has long been documented in the UK and continental Europe (Lacey *et al.*, 1987) as well as OC (Cheah *et al.*, 1982). Isolates of complementary *MAT1‐1* and *MAT1‐2* types are required for sexual reproduction (Ilott *et al.*, 1984; Foster *et al.*, 2002). Apothecia have not been found in association with outbreaks of light leaf spot in NA, and it is not known whether a sexual cycle occurs in NA. However, this information is important to underpin management strategies for light leaf spot, as populations with both sexual and asexual reproduction tend to have greater evolutionary potential than those that are exclusively asexual (McDonald and Linde, 2002). Such populations also present a greater risk of failures in disease management strategies, for example, if strains of the pathogen overcome host resistance genes (Boys *et al.*, 2007) or develop resistance to fungicides commonly used in brassica crops, as has occurred in the UK and continental Europe (Carter *et al.*, 2013, 2014).

Effective management of light leaf spot in areas where this disease has established has necessitated the integration of planting cultivars with resistance to the disease, applying fungicides with efficacy against the pathogen, and implementing cultural practices such as incorporation of infected crop residues into the soil and/or crop rotation (Karandeni Dewage *et al.*, 2018). Host resistance alone has been insufficient to control economically damaging outbreaks of light leaf spot in *B. napus* crops as there are no fully resistant commercial cultivars currently available (Boys *et al.*, 2007, 2012). Thus, management of this disease in conventional crops has depended on applications of fungicides, including methyl benzimidazole carbamates (MBCs, Fungicide Resistance Action Committee [FRAC] Group 1) and azoles (sterol 14α‐demethylation inhibitors [DMIs], FRAC Group 3; Carter *et al.*, 2013, 2014). However, reduced sensitivity to these fungicides has been reported for some UK and continental European isolates of *P. brassicae*, and the molecular mechanisms of resistance have been characterized (Carter *et al.*, 2013, 2014). Genotypic and phenotypic data on fungicide sensitivity of NA isolates of the light leaf spot pathogen are needed to monitor the current and future potential efficacy of fungicide applications for control of this disease in NA.

Given the increasing losses associated with light leaf spot in areas where this disease is well established, and preliminary evidence of genetic differentiation of isolates of the fungus causing this disease in NA from isolates in the UK and continental Europe, there is a need to characterize these pathogen populations. The primary objective of this study was to compare isolates of the light leaf spot pathogen from regions where *P. brassicae* has long been established, i.e., the UK and continental Europe and OC (Majer *et al.*, 1998), with isolates from NA, where light leaf spot was found recently. The isolates evaluated in this study were obtained from a range of Brassicaceae genera and species, and compared using the consolidated species concept (CSC) by combining morphological, ecological, biological, and genetic (phylogenetic) data (Crous *et al.*, 2015).

## 2 MATERIALS AND METHODS

### 2.1 *Pyrenopeziza* isolates and herbarium specimens

Details of the light leaf spot fungal isolates used in this study, including isolates and herbarium specimens of infected leaves submitted to the Westerdijk Fungal Biodiversity Institute (WFBI) in the Netherlands, isolates deposited in the International Mycological Institute (IMI) collection in the UK, and GenBank accession numbers for fungal DNA sequences, are listed in Table 1. The GenBank accession numbers listed in Table 1 were all generated as part of this study except for the following: OC isolates were obtained from the WFBI (CBS157.35) or the IMI herbarium (IMI233715 to IMI233717), and the older isolates from the UK or continental Europe (preceding 2000) were obtained from collectors or programmes listed in Table 1. For each of the UK or continental Europe isolates generated in this study, infected leaves from a collection at Rothamsted Research were examined with a stereomicroscope, and a single pustule was placed into a drop of sterile distilled water (SDW) using a sterilized needle. The conidial suspension was spread onto a plate of 3% malt extract agar (MEA) using a sterilized disposable loop, and incubated at 15 °C for 10 days. Single colonies were then used to establish single‐spore cultures. For each NA isolate, small pieces (up to 5 mm^2^) of leaf and stem tissue with symptoms were surface‐sterilized in 1.2% NaOCl for up to 2 min, and rinsed three times in SDW; or sterilized in 70% ethanol for 5 s, dried on sterilized blotter paper, and plated onto clarified V8 (cV8) agar amended with chloramphenicol (100 mg/L; Carmody, 2017). The leaf pieces were incubated under a day/night cycle of 15 °C with cool white fluorescent light and near‐ultraviolet (NUV) light for 8 hr/day, and 10 °C in the dark for 16 hr/day. The cultures were used to generate single‐spore isolates by streaking a spore suspension of each isolate onto water agar (WA) and picking individual colonies. Single‐spore isolates were maintained in 88% glycerol suspensions at −80 °C in the Rothamsted Research (UK) culture collection, and at the Washington State University (WSU) Mount Vernon Northwestern Washington Research & Extension Center (NWREC) on dried, colonized filter disks stored at −20 °C with desiccant.

**Table 1:** Isolate accession numbers and herbarium accession numbers for infected turnip leaves submitted to the Westerdijk Fungal Biodiversity Institute (WFBI), International Mycological Institute (IMI) isolate accession numbers, and GenBank DNA sequence accession numbers for isolates of *Pyrenopeziza* associated with light leaf spot of brassicas in the United Kingdom, continental Europe, Oceania, and North America that were evaluated in this study

Continent/isolate code (lineage)	Isolate origin	Year collected	Original host *Brassica* or *Raphanus* species	*MAT* type^b^	Original collector	WFBI herba‐rium accession no.	WFBI live culture accession no.	IMI live culture accession no.	GenBank accession no. of DNA region or gene^a^			
									ITS rDNA	*β‐tubulin*	*TEF1‐α*	*MAT*
Continental European (EU) or United Kingdom (UK; Lineage 1)												
PC13	Rostock, Germany, EU	1995	*B. napus*	*MAT1‐1*	D. Majer				MF187545	MF314352	MF314381	
PC17	Cambridge, UK	1994	*B. napus*	*MAT1‐2*	D. Majer				MF187536	MF314353	MF314380	
PC18	Aberdeen, UK	1994	*B. napus*	*MAT1‐2*	D. Majer				MF187547	MF314354	MF314379	
PC19	Rostock, Germany, EU	1995	*B. napus*	*MAT1‐1*	D. Majer				MF187546	MF314355	MF314378	MF314436
PC20	Edinburgh, UK	1994	*B. napus*	*MAT1‐2*	D. Majer				MF187539	MF314356	MF314377	
PC22	Cambridge, UK	1994	*B. napus*	*MAT1‐2*	D. Majer				MF187535	MF314357	MF314376	
PC23	Rostock, Germany, EU	1995	*B. napus*	*MAT1‐1*	D. Majer				MF187543	MF314358	MF314375	MF314432
PC28	Edinburgh, UK	1994	*B. napus*	*MAT1‐1*	D. Majer				MF187538	MF314359	MF314374	MF314437
PC30	Cambridge, UK	c. 1994	*B. napus*	*MAT1‐2*	D. Majer				MF187531	MF314360	MF314373	MF314417
PC32	Cambridge, UK	1994	*B. napus*	*MAT1‐2*	D. Majer				MF187537	MF314361	MF314372	MF314418
PC35	Le Rheu, France, EU	1995	*B. napus*	*MAT1‐1*	D. Majer				MF187534	MF314362	MF314371	MF314430
PC38	Cambridge, UK	c. 1994	*B. napus*	*MAT1‐2*	D. Majer				MF187544	MF314363	MF314370	MF314419
PC39	Aberdeen, UK	1994	*B. napus*	*MAT1‐1*	D. Majer				MF187541	MF314364	MF314369	MF314433
PC45	Yorkshire, UK	1996	*B. oleracea*	*MAT1‐2*	P. Gladders				MF187542	MF314365	MF314368	MF314420
PC50	Aberdeen, UK	1998	*B. napus*	*MAT1‐1*	D. Majer				MF187540	MF314366	MF314367	MF314434
17KALE02	Lincolnshire, UK	2017	*B. oleracea* (kale)	*MAT1‐1*	K. M. King			IMI506783				
2016‐5 (S, CO)^c^	Northumberland, UK	2016	*B. napus*	*MAT1‐2*	N. J. Hawkins	CBS23334	CBS143753	IMI506784				MF314404
2016‐9 (S, M, CO)^c^	Northumberland, UK	2016	*B. napus*	*MAT1‐1*	N. J. Hawkins	CBS23335	CBS143754	IMI506785				MF314442
2016‐26 (S, CO)	Northumberland, UK	2016	*B. napus*	*MAT1‐1*	N. J. Hawkins	CBS23336	CBS143755					MF314441
2016‐34 (S, CO)	Northumberland, UK	2016	*B. napus*	*MAT1‐1*	N. J. Hawkins	CBS23337	CBS143756	IMI506787				
2016‐50 (S, M, CO)	Northumberland, UK	2016	*B. napus*	*MAT1‐2*	N. J. Hawkins	CBS23338	CBS143757	IMI506788				MF314405
4e	Northumberland, UK	2013	*B. napus*	*MAT1‐1*	N. J. Hawkins				MF187532	MF314350	MF314394	MF314431
5a (S, CO)	Northumberland, UK	2013	*B. napus*	*MAT1‐2*	N. J. Hawkins	CBS23339	CBS143758	IMI506781	MF187533	MF314362	MF314371	MF314430
Pb12	Scotland, UK	2008	*B. napus*	No data	J. A. Lucas							
8CAB (S, M, CO)	East Lothian, UK	2011	*B. oleracea* (broccoli)	*MAT1‐1*	P. Gladders	CBS23340	CBS143759	IMI506782				
E3A (S, CO)	Hertfordshire, UK	2007	*B. napus*	*MAT1‐2*	E. Boys	CBS23341	CBS143760	IMI506798				MF314407
FR2 (S, M, CO)	Le Rheu, France, EU	1995	*B. napus*	*MAT1‐1*	D. Majer	CBS23342	CBS143761	IMI506799				—
JT2A– (S)	Hertfordshire, UK	2009	*B. rapa* (turnip rape)	*MAT1‐2*	J. S. West							MF314412
UK73 (S, CO)	Angus, UK	2005	*B. napus*	*MAT1‐2*	No data	CBS23343	CBS143762	IMI506800				MF314421
IMI204290	Oxfordshire, UK	1975	*B. napus*	*MAT1‐2*	C. J. Rawlinson							MF314408
IMI81823^d^	Worcestershire, UK	1956	*B. oleracea*	No data	C. J. Hickman				MN028386			
Oceania (Lineage 1)												
CBS157.35	Victoria, Australia	1935	*B. oleracea*	*MAT1‐1*	E. McLennan				MH855615			MF314438
IMI233715	New Zealand	1978	*B. oleracea*	*MAT1‐2*	W. F. Harthill, C. J. Rawlinson							MF314409
IMI233716	New Zealand	1978	*B. oleracea*	*MAT1‐2*	W. F. Harthill, C. J. Rawlinson				MF187548	MF314351	MF314395	MF314410
IMI233717	New Zealand	1978	*B. oleracea*	*MAT1‐2*	W. F. Harthill, C. J. Rawlinson							MF314411
North America (Lineage 2)												
Cyc001 (S, M, CO)	Benton Co., OR, USA	2015	*B. rapa* (Barkant turnip)	*MAT1‐2*	S. M. Carmody	CBS23324	CBS143743	IMI506789	MF143610	MF314337	MF314392	MF314396
Cyc007	Skagit Co., WA, USA	2016	*B. rapa* (birds‐rape mustard)	*MAT1‐2*	S. M. Carmody			IMI506790	MF143611	MF314338	MF314391	MF314397
Cyc009A (M, CO)	Mount Vernon, Skagit Co., WA, USA	2016	*B. rapa* (birds‐rape mustard)	*MAT1‐2*	S. M. Carmody	CBS23325	CBS143744		MF143613	MF314339	MF314390	MF314398
Cyc011A (M, CO)	Edison, Skagit Co., WA, USA	2016	*B. rapa* (birds‐rape mustard)	*MAT1‐1*	S. M. Carmody	CBS23326	CBS143745	IMI506791	MF143615	MF314340	MF314389	MF314425
Cyc013A (M, CO)	Skagit Co., WA, USA	2016	*B. rapa* (birds‐rape mustard)	*MAT1‐2*	S. M. Carmody	CBS23327	CBS143746	IMI506792	MF143617	MF314341	MF314388	MF314399
Cyc015 (M, CO)	Skagit Co., WA, USA	2016	*B. juncea* (mustard cover crop)	*MAT1‐1*	S. M. Carmody	CBS23328	CBS143747	IMI506793	MF143619	MF314342	MF314387	MF314422
Cyc017 (M, CO)	Skagit Co., WA, USA	2016	*B. rapa* (birds‐rape mustard)	*MAT1‐1*	S. M. Carmody	CBS23329	CBS143748	IMI506794	MF143620	MF314343	MF314386	MF314423
Cyc023A (M, CO)	Corvallis, Benton Co., OR, USA	2016	*B. rapa* (Purple top globe turnip)	*MAT1‐1*	L. J. du Toit	CBS23330	CBS143749		MF143621	MF314344	MN044437	MF314424
Cyc024A	Whatcom Co., WA, USA	2016	*B. rapa*	*MAT1‐2*	S. M. Carmody	CBS23331	CBS143750		MF143622	MF314345	MF314385	MF314400
Cyc025 (M, CO)	Snohomish Co., WA, USA	2016	*B. rapa* (birds‐rape mustard)	*MAT1‐2*	S. M. Carmody	CBS23332	CBS143751	IMI506796	MF143623	MF314346	MF314384	MF314401
Cyc029 (M, CO)	Snohomish Co., WA, USA	2016	*B. rapa* (birds‐rape mustard)	*MAT1‐2*	S. M. Carmody	CBS23333	CBS143752		MF143627	MF314347	MF314383	MF314402
Cyc031	Corvallis, Benton Co., OR, USA	2016	*B. rapa*	No data	L. J. du Toit				MK995633	MF314349	MF314382	
14CC2B (M, CO)	Polk Co., OR, USA	2014	*B. napus* (canola)	*MAT1‐1*	B. Claassen							MF314426
14CC4A	Polk Co., OR, USA	2014	*B. napus* (canola)	*MAT1‐1*	B. Claassen							MF314427
14CC8A	Polk Co., OR, USA	2014	*Raphanus* sp. (wild radish)	*MAT1‐1*	B. Claassen							MF314428
15LS13B	Benton Co., OR, USA	2015	*B. juncea* (red mustard)	*MAT1‐1*	B. Claassen							MF314429
223	Douglas Co., OR, USA	2016	*B. rapa* (birds‐rape mustard)	*MAT1‐2*	B. Claassen							MF314403

a ITS rDNA = internal transcribed spacer (ITS) region of ribosomal DNA (rDNA); *β‐tubulin* = *β‐tubulin* gene; *TEF1‐α* = *translation elongation factor 1‐α* gene; *MAT* = mating type genes of the light leaf spot pathogen (Ilott *et al*., 1984; Foster *et al*., 2002). All sequences with accession numbers in this table were generated in this study.

b Isolates confirmed as *MAT1‐1* or *MAT1‐2* type using the multiplex PCR assays of Foster *et al*. (2002). All mating type sequences with accession numbers in this table were generated as part of this study.

c S, isolates from continental Europe and UK (*n* = 10) inoculated onto *Brassica rapa* ‘Hakurei’ to compare symptomology with that caused by North American isolate Cyc001, as detailed in the main text. M = isolates from continental Europe and UK (*n* = 4) compared with isolates from North America (*n* = 10) for morphology on malt extract agar, as detailed in the main text. CO, isolates used to compare conidial morphology in vitro and in vivo, as detailed in the main text.

d Type specimen of *P. brassicae* examined in the form of apothecia in dried culture (Rawlinson *et al*., 1978). Only a partial ITS rDNA sequence (MN028386) could be amplified from the herbarium specimen.

### 2.2 DNA extraction

At Rothamsted Research, genomic DNA was extracted from lyophilized mycelium of each isolate using a MasterPure Yeast DNA Purification kit (Epicentre). DNA concentration was then quantified using a NanoDrop spectrophotometer, and diluted to the required concentration using PCR‐grade water. At the WSU Mount Vernon NWREC, genomic DNA was extracted from mycelium harvested from potato dextrose broth liquid cultures using a DNeasy Plant Mini Kit (QIAGEN). DNA concentration was then quantified using a Qubit fluorometer, and diluted to the required concentration using PCR‐grade water.

### 2.3 Genus confirmation and multilocus sequence analysis

**Figure 1:**
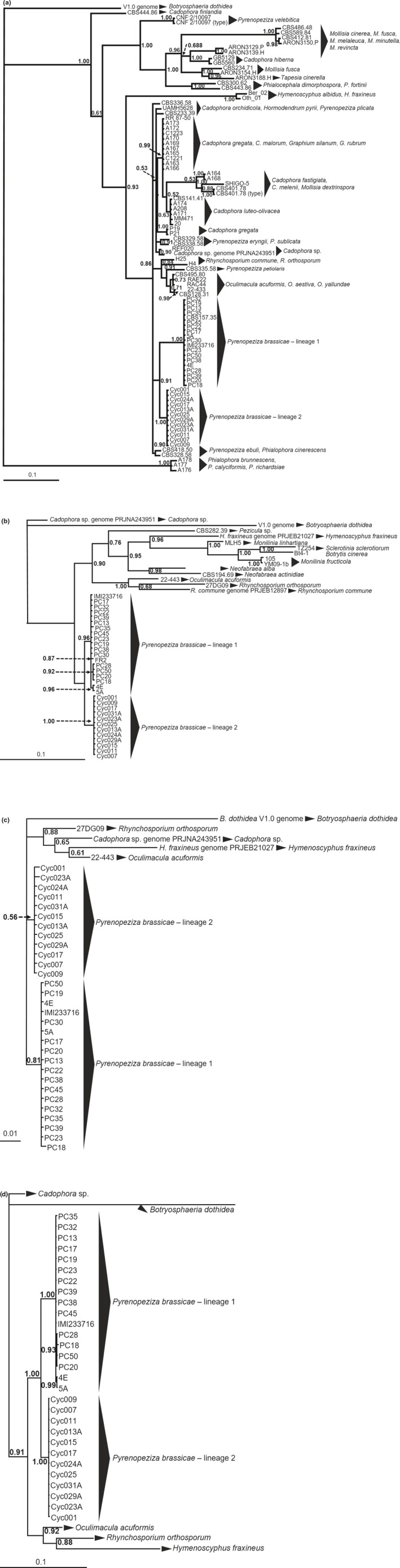
Phylogenetic trees from Bayesian analysis of multiple gene sequences obtained from *Pyrenopeziza brassicae* isolates from the United Kingdom (UK), continental Europe (EU), North America (NA), and Oceania (OC), as well as other fungal genera and species. Trees were constructed with partial sequences from (a) the internal transcribed spacer (ITS) region of ribosomal DNA (rDNA); (b) the *β‐tubulin* gene; (c) the *translation elongation factor1‐α* (*TEF1‐α*) gene; and (d) the concatenated sequences from all three regions. Bayesian posterior probabilities are indicated at the nodes (BPP). The outgroup sequence used for each analysis was from *Botryosphaeria dothidea.* Refer to Table 1 and Table S1 for details of the isolates and sequences

To verify identity of the genus of the NA isolates as *Pyrenopeziza*, phylogenetic analyses were completed for the partial ITS rDNA of 30 isolates of the light leaf spot pathogen (12 from NA, 13 from the UK, 4 from continental Europe, and 2 from OC) along with ITS rDNA sequences of isolates of 57 related fungi, including sequences available in GenBank for seven other *Pyrenopeziza* species (*P. ebuli*, *P. eryngii*, *P. petiolaris*, *P. plicata*, *P. revincta*, *P. subplicata*, and *P. velebitica*), nine *Cadophora* species, two *Graphium* species, *Hormodendrum pyri*, two *Hymenoscyphus* species, *Leptodontidium orchidicola*, five *Mollisia* species, three *Oculimacula* species, four *Phialophora* species, two *Phialocephala* species, two *Rhynchosporium* species, and *Tapesia cinerella* (Table 1; Table S1; Figure 1a). The ITS rDNA sequence obtained from a genome of *Botryosphaeria dothidea* served as the outgroup (Table S1). In addition, the *β‐tubulin* and *translation elongation factor 1‐α* (*TEF1‐α*) genes were amplified from the same 30 isolates of *P. brassicae* isolates from the UK and continental Europe, OC, and NA, as well as from closely related fungi (Table 1; Table S1), for completing individual phylogenetic analyses of each DNA region as well as multilocus sequence analysis (MLSA) of concatenated sequences of the three DNA regions. Relevant sequences from *B. dothidea* served as outgroups for these analyses (Crous *et al.*, 2003; Table S1; Figure 1b–d).

Primers used for the amplification of various DNA sequences are detailed in Table 2. The ITS rDNA was amplified as described by Bakkeren *et al*. (2000) in a total reaction volume of 30 μl that included 1× buffer (Invitrogen Life Technologies), 1.5 mM MgCl_2_, 0.2 mM of each dNTP, 0.4 mM of each primer, 1.5 U *Taq* DNA polymerase (Invitrogen Life Technologies), and 2 μl genomic DNA. The *β‐tubulin* gene was amplified as detailed by Einax and Voigt (2003) in a total reaction volume of 25 μl, including 1× buffer, 1.5 mM MgCl_2_, 0.4 mM of each dNTP, 0.24 mM of each primer, 1.25 U *Taq* DNA polymerase, and 1 μl genomic DNA. The *TEF1‐α* gene was amplified using the protocol described by Taşkin *et al*. (2010) in a total reaction volume of 20 μl, which included 1× buffer, 1.5 mM MgCl_2_, 0.15 mM of each dNTP, 0.15 mM of each primer, 1 U *Taq* DNA polymerase, and 2 μl genomic DNA. PCRs were done in a Thermohybaid PCR Express thermocycler (ThermoFisher Scientific) using the following cycles: 94 °C for 3 min; 31 cycles of 92 °C for 45 s, 60 °C for 45 s, and 72 °C for 1 min; and 72 °C for 10 min for ITS rDNA amplification; 94 °C for 3 min; 35 cycles of 92 °C for 45 s, 55 °C for 45 s, 72 °C for 1 min; and 72 °C for 10 min for *β‐tubulin* amplification; and 95 °C for 2 min; 35 cycles of 95 °C for 15 s, 58 °C for 45 s, and 72 °C for 45 s; and 72 °C for 5 min for *TEF1‐α* amplification.

**Table 2:** Primers used in PCR assays to amplify the internal transcribed spacer (ITS) ribosomal DNA (rDNA) region, *β‐tubulin* gene, *TEF1‐α* gene, *MAT1‐1‐3* gene, and *MAT1‐2‐1* gene of isolates of *Pyrenopeziza* from the United Kingdom, continental Europe, Oceania, and North America that were associated with light leaf spot of brassicas, for phylogenetic comparisons of isolates from these geographic regions

DNA target	Primer name	Sequence (5′–3′)	Reference
ITS rDNA	Forward primer UNUP18S42	CGTAACAAGGTTTCCGTAGGTGAAC	Bakkeren *et al*. (2000)
	Reverse primer UNLO28S576B	GTTTCTTTTCCTCCGCTTATTAATATG	
*β‐tubulin*	Forward primer F‐Btub3	TGGGCYAAGGGTYAYTAYAC	Einax and Voigt (2003)
	Reverse primer F‐Btub2r	GGRATCCAYTCRACRAA	
*TEF1‐α*	Forward primer EF5AR	CCAGCAACRTTACCACGACG	Taşkin *et al*. (2010)
	Reverse primer EF2F	AACATGATSACTGGTACYTCC	
*MAT1‐1* and *MAT1‐2*	PbM‐1‐3	GATCAAGAGACGCAAGACCAAG	Foster *et al*. (2002)
	PbM‐2	CCCGAAATCATTGAGCATTACAAG	
	Reverse primer Mt3	CCAAATCAGGCCCCAAAATATG	

Note

Refer to the main text for details of each PCR assay, and to Table 1 for details of the fungal isolates used for each PCR assay.

After running the amplified products on 1.5% agarose gels to confirm single bands, PCR products were cleaned using an ExoSAP‐IT kit (ThermoFisher Scientific) and sent to Elim Biopharmaceuticals, Inc. for bidirectional sequencing. Primers used for PCR amplification were also used in the sequencing reactions (Table 2). The DNA sequences were processed using MEGA v. 7 (Kumar *et al.*, 2016), and deposited in GenBank (Table 1).

#### 2.3.1 Phylogenetic analysis

Partial sequences from the ITS rDNA region, *β‐tubulin* gene, and *TEF1‐α* gene, along with concatenated sequences of the three regions were aligned using ClustalW in Geneious v. 10.2.3 (Biomatters Ltd.), and trimmed to equal lengths of 485 nt for the ITS rDNA, 662 nt for *β‐tubulin*, and 535 nt for *TEF1‐α*. Model selection was done using jModelTest v. 2.1.1.0 (Darriba *et al.*, 2012).

Bayesian analyses were completed using MrBayes v. 3.2.6 (x64). The Markov chain Monte Carlo (MCMC) analyses for individual genes and the concatenated alignment were run for 10^6^ generations, with the first 25% discarded in the initial burn‐in and chains subsampled every 500 generations. The best‐fit model used for each analysis was GTR + I + G, except for the *TEF1‐α* gene for which the GTR + G model was selected. The MCMC output was inspected to confirm acceptable burn‐in length and chain convergence (stationarity), and the consensus trees were viewed in TreeView v. 1.6.6. The phylogenetic trees for individual DNA sequences and the concatenated sequences (Figure 1) were submitted to Treebase (TB2:S24431). In addition, maximum‐likelihood analyses were completed with the same ClustalW alignments as for the Bayesian analyses, using the PhyML (3.3.20180621) plug‐in in Geneious. For all analyses, the GTR model was selected and bootstrapping was based on 100 replications. The consensus trees were rooted with *B. dothidea* sequences and viewed using TreeView.

### 2.4 Mating type screening, distribution, and phylogeny

Sequences of the *MAT1‐1* and *MAT1‐2* genes were amplified from 40 isolates of *P. brassicae* (Table 1) to enable phylogenetic analyses of these mating type genes. Sequences were obtained from the isolates (Table 1) using the Foster *et al*. (2002) multiplex PCR assay. Reactions were done in 20 µl volumes, each containing 10 µl MegaMix‐Blue (Microzone); 1 µl each of primers PbM‐1‐3, PbM‐2, and the reverse primer Mt3 (Table 2), with each primer at a final concentration of 0.5 µM; 5 µl PCR grade water; and 2 µl unquantified DNA extract. Amplicons were resolved on a 2% agarose gel and sent to MWG Eurofins for sequencing with primer Mt3.

### 2.5 Rep‐PCR DNA fingerprinting

Rep‐PCR fingerprinting of a selection of nine isolates of the light leaf spot pathogen from NA and 10 isolates from the UK, continental Europe, and OC was done using the protocols and primers described by Versalovic *et al*. (1994). Each reaction was completed in a 20 µl volume containing 10 µl JumpStart REDTaq ReadyMix (Sigma Aldrich), 2–4 µl of each primer (see details below), 6 µl PCR‐grade water, and 2 µl DNA (20 ng total per reaction). Three variants of rep‐PCR fingerprinting were done: (a) BOX PCR for which each reaction included 4 µl of primer BOXAIR at a 1 µM final concentration; (b) ERIC PCR for which each reaction included 2 µl each of primers ERIC1R/ERIC2 with each primer at a 0.5 µM final concentration; and (c) GTG^5^ PCR for which each reaction included 4 µl of primer GTG^5^ at a 1 µM final concentration. Reaction conditions were: 96 °C for 2 min; 35 cycles of 94 °C for 30 s, 52 °C for 1 min, and 65 °C for 5 min; and a final step at 65 °C for 8 min. PCR products (8 µl) were subsequently visualized on a 2% agarose gel (110 V for 3 hr) with ethidium bromide.

### 2.6 Pathogenicity of NA isolates

*Brassica rapa* turnip plants (cv. Hakurei; Osborne International Seed Co.) and *B. juncea* mustard plants (cv. Caliente 199; High Performance Seeds, Inc.) were used to test pathogenicity of 17 NA isolates of the light leaf spot pathogen (Table 1). Seeds of each cultivar were sown in RediEarth Seedling Starter Mix (SunGro) in 72‐cell flats (two seeds per cell, with each cell 3.8 cm diameter × 5.7 cm deep) in a greenhouse at 20 ± 3 °C by day and 15 ± 3 °C by night with supplemental lighting for 12 hr/day, at the WSU Mount Vernon NWREC. Three weeks later, the seedlings were transplanted into Sunshine Mix #1 (SunGro) in 15 cm diameter plastic pots. Plants were inoculated with the light leaf spot isolates 6 weeks after transplanting. The day prior to inoculation, the plants were incubated overnight in polyethylene bags under a greenhouse bench that was covered with two layers of Remay cloth for shading to prevent plants overheating in the bags.

Based on limited availability of space, the 17 NA isolates were tested for pathogenicity in groups over a total of three trials (four isolates in Trial 1, two isolates in Trial 2, and 11 isolates in Trial 3) at the WSU Mount Vernon NWREC (Table 1). A conidial suspension was prepared for each isolate using 6‐ to 8‐week‐old colonized plates of V8 agar medium by adding 20 ml SDW onto the surface of each plate and gently rubbing the surface of the culture using a sterilized, bent glass rod. Each spore suspension was filtered through two layers of cheesecloth, and the concentration adjusted to 10^6^ conidia/ml, to which 0.01% Tween 20 was added. Four replicate plants each of *B. rapa* and *B. juncea* were inoculated with either: (a) a tester NA isolate; (b) a NA isolate previously demonstrated to be pathogenic on brassicas (Cyc001, the positive control treatment); or (c) SDW (negative control treatment). Each treatment was applied using an atomizer (Rescende Model 175, Badger Air‐Brush Co.) until the leaves were coated with fine droplets. Plants were then placed back in the polyethylene bags under greenhouse benches covered in Remay for 48 hr to promote fungal infection, removed from the bags, and laid out on greenhouse benches in a randomized complete block (RCB) design.

Each inoculation trial was set up as a two‐factor factorial treatment design consisting of the two *Brassica* species (*B. juncea* and *B. rapa*) inoculated with the test isolates and control treatments. Three leaves of each plant were rated 14 and 21 days after inoculation (dai) for the type of symptoms (chlorosis and/or necrosis) and the percentage of leaf area with symptoms. Those plants on which veinal browning was the primary symptom were rated as having 1% symptom severity. The mean severity ratings of three leaves per plant for each replication of each treatment combination were subjected to analysis of variance (ANOVA), with replication treated as a random effect, and plant species and isolates as fixed effects. Data from the SDW‐treated control plants were excluded from the ANOVA because symptoms did not develop on those plants. Assumptions of normality and equal variance were tested. Treatment means were compared using Fisher's protected least significant difference (LSD) at *p* < .05. Lesions that developed were examined microscopically 21 dai to confirm the presence of acervuli and conidia of the pathogen. Isolations from lesions caused by each of the 17 isolates were completed as described above for the original light leaf spot samples collected in NA, and ITS rDNA and *β‐tubulin* sequences were generated from the reisolates as described above.

**Figure 2:**
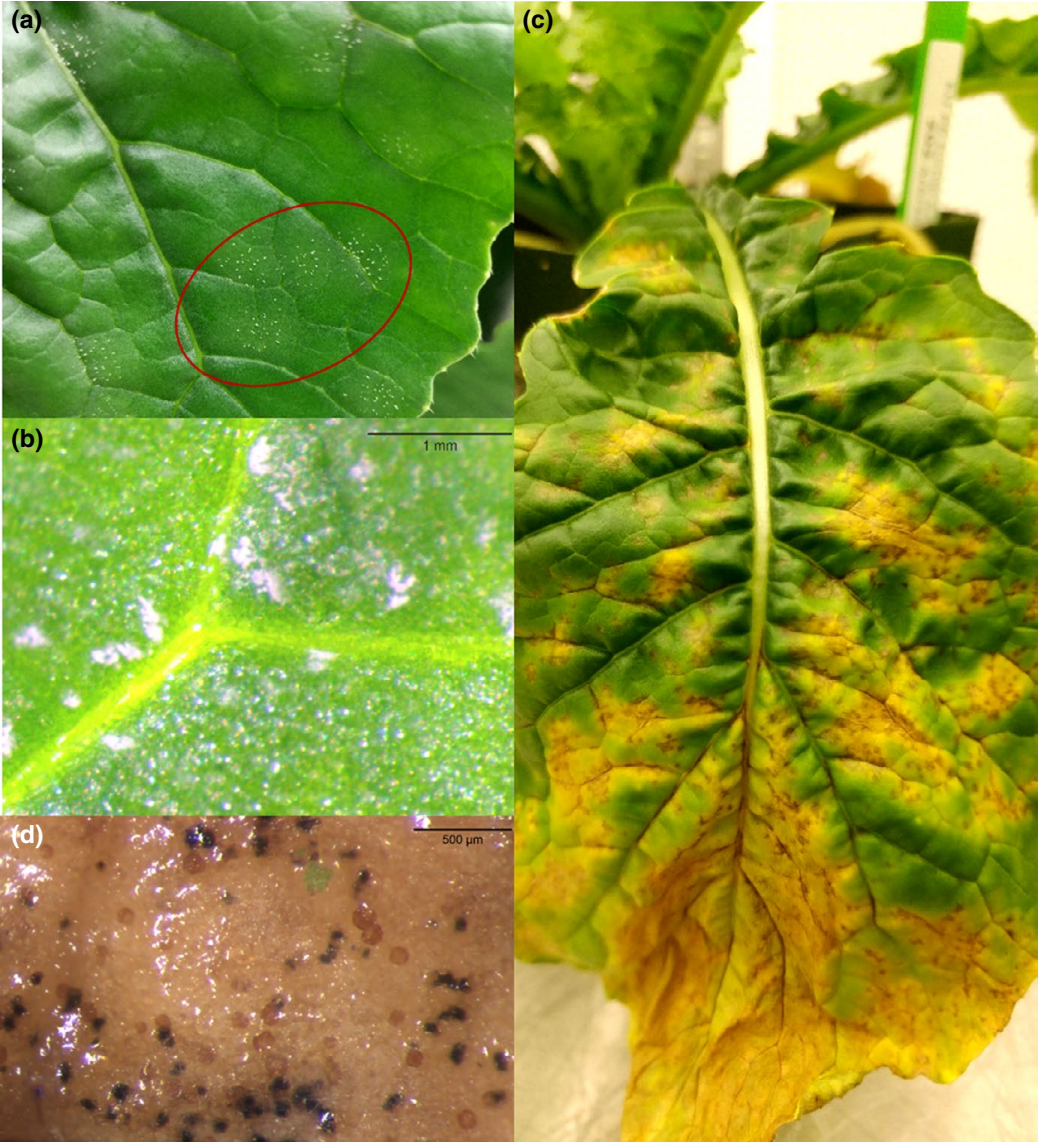
(a, b) Light leaf spot signs (patches of white conidiomata) produced by isolate 2016‐26 of *Pyrenopeziza brassicae* from the United Kingdom (Lineage 1), 14 days after inoculation (dai) of Hakurei turnip (*Brassica rapa*) plants. (b) Close‐up image of white conidiomata produced by 2016‐26, a Lineage 1 isolate of *P. brassicae* on a turnip leaf. (c) Symptoms of light leaf spot caused by isolate Cyc001 (Lineage 2) of *P. brassicae* from Benton Co. 21 dai, were typical of those observed for other isolates collected in Washington and Oregon, i.e., coalescing chlorotic spots and veinal browning without any white conidiomata. (d) Typical pale tan to brown, circular acervuli and black stromatal knots observed on turnip leaves infected with Cyc001, a Lineage 2 North American isolate, after incubating the leaf section on V8 agar medium on a laboratory bench at room temperature for approximately 7 days

To compare symptoms caused by isolates from the UK and continental Europe versus isolates from NA, *B. rapa* turnip seedlings (cv. Hakurei) were grown in a greenhouse as described above. Four replicate plants were inoculated with each of 11 light leaf spot isolates (10 from the UK and continental Europe as well as NA isolate Cyc001) or SDW as described above, with the plants laid out in a RCB design. Plants were scored for the presence or absence of circular patches of white conidiomata on three leaves per plant at 28 dai (Figure 2a,b). The number of inoculated leaves that were chlorotic, necrotic (senesced), or had patches of white conidiomata were rated 21 and 28 dai (based on the total number of leaves present at the time of inoculation). Reisolations of fungi were done from leaf spot lesions for the NA isolate, from sections of leaves with white conidiomata for UK and continental Europe isolates, or from symptomless tissue for control plants treated with SDW, as described previously. In addition, leaf sections were examined microscopically for *Pyrenopeziza* acervuli and conidia.

Leaf rating data were subjected to ANOVA for the number of inoculated leaves with white conidiomata per plant, the number of inoculated necrotic leaves per plant, and the number of inoculated chlorotic leaves per plant 28 dai. Replications were treated as a random effect and isolates as a fixed effect in the model. Control plants treated with SDW were excluded from the analyses because symptoms did not develop on those plants. Plants inoculated with the NA isolate were excluded from the ANOVA for the number of inoculated leaves with white conidiomata, as none was observed on those plants. Disease severity ratings 28 dai were used for ANOVAs because the number of necrotic leaves was much greater than at 21 dai. Assumptions of normality and equal variance were tested. Assumptions for parametric analysis were met for the number of inoculated leaves with white conidiomata and the number of inoculated leaves that turned necrotic, while data for the number of inoculated leaves that turned chlorotic had to be analysed using Friedman's nonparametric rank test. Treatment means were compared using Fisher's protected LSD at *p* < .05. The pathogenicity test was repeated.

### 2.7 Sexual compatibility testing

Twenty light leaf spot isolates, 10 from NA (five *MAT1‐1* and five *MAT1‐2*) and 10 from the UK or continental Europe (five *MAT1‐1* and five *MAT1‐2*), were grown from −80 °C glycerol stocks onto 3% MEA plates, incubated in the dark at 18 °C, and used to attempt sexual crosses (Tables 1 and 3). After 6 weeks, 1 ml SDW water was added to the surface of each stock plate and the colonies agitated using a sterilized bent glass rod. The conidial suspension was filtered through a double layer of sterilized cheesecloth and adjusted to 10^6^ conidia/ml. A 40 µl aliquot of conidial suspension from each of the two isolates used for each attempted sexual cross was placed onto a plate of 3% MEA and the two aliquots spread across the agar surface using a sterilized bent glass rod. Plates were sealed with Parafilm and incubated for a further 9 weeks in the dark at 18 °C, after which plates were examined microscopically at weekly intervals for the presence or absence of apothecial initials, mature apothecia, and asci with ascospores (the latter determined microscopically from thin apothecial sections examined at ≤100× magnification). Each sexual cross was attempted using three replicate plates of MEA.

**Table 3:** Attempted sexual crosses of isolates of *Pyrenopeziza brassicae* (Lineage 1) from the United Kingdom and continental Europe (EU) with isolates (Lineage 2) from North American (NA) associated with light leaf spot, using isolates of opposite mating (*MAT*) type paired on 3% malt extract agar

	Lineage	Isolate	*MAT1‐1* type^a^									
			EU and UK isolates (Lineage 1)					NA isolates (Lineage 2)				
			2016‐9	2016‐26	2016‐34	8CAB	FR2	14CC2	Cyc011A	Cyc015	Cyc017	Cyc023A
*MAT1‐2* type^a^	EU & UK isolates (Lineage 1)	2016‐5	As^b^	As^3^	As^3^	As^3^	As^1^	−	−	Ai	Ai	Ai
		2016‐50	Ap^1^	−	As^1^	As^2^	−	−	−	−	−	−
		5a	As^3^	As^2^	As^3^	As^2^	As^2^	Ai	−	‐	Ai	−
		E3A	As^3^	As^3^	Ai^1^, As^2^	As^2^	As^2^	−	−	Ai	−	−
		UK73	Ai^1^, Ap^1^	As^2^	Ap^1^	Ap^1^, As^2^	Ai^1^	−	−	−	−	−
	NA isolates (Lineage 2)	Cyc001	−	Ai	−	Ai	−	−	−	−	−	−
		Cyc009A	Ai	−	Ai	Ai	−	−	−	−	−	−
		Cyc013A	−	−	−	−	−	−	−	−	−	−
		Cyc025	−	−	−	−	−	−	−	−	−	−
		Cyc029A	−	−	−	−	−	−	−	−	−	−

a Isolates were confirmed as either *MAT1‐1* or *MAT1‐2* types using the multiplex PCR assays of Foster *et al*. (2002).

b Three replicate pairings were established for each attempted sexual cross. The superscript number denotes the number of replicate plates on which apothecial initials (Ai), apothecia (Ap), or asci and ascospores (As) were observed. ‘−’ indicates no sexual structures were observed. Results shown were after the isolates had been paired on 3% malt extract agar for 9 weeks. Refer to Table 1 for details of each isolate.

### 2.8 Morphological comparison

Light leaf spot isolates from NA and from the UK and continental Europe were compared morphologically in vitro and in planta (Table 1). For in vitro comparison, cultures were initiated from −80 °C glycerol stocks onto three replicate 3% MEA plates for each of four isolates from the UK and continental Europe compared to 10 NA isolates. The plates were incubated at 18 °C in the dark for 4 months, at which time the plates were photographed. For comparison of conidial morphologies in vitro, 10 UK and continental Europe isolates, and eight NA isolates (all isolates listed in Table 3, excluding two of the 10 NA isolates which sporulated poorly) were grown for 6 weeks on 3% MEA as detailed above, after which conidia were harvested and examined microscopically. Conidial shape was examined for each isolate, and the length and diameter of 25 conidia per isolate were measured using a digital CCD camera (Hamamatsu C8484 05G01) and HCimage software (Hamamatsu Photonics K.K.). Conidial dimensions for the UK and continental European isolates were compared with those of the NA isolates using Student's *t* test (Graphpad Software).

For examination of conidial morphology in planta, conidia were washed from inoculated leaves with symptoms from *B. rapa* turnip (cv. Hakurei) plants that had been inoculated 28 days previously with 20 isolates of the light leaf spot pathogen (10 continental Europe and UK isolates, and 10 NA isolates; Table 1). The length and width, and the presence or absence of a septum, were recorded for each of 60 conidia per isolate. Photographs of conidia were taken with a Leica camera (DFC295, Wetzlar) and Leica Application Software v. 3.8 (Leica Microsystems). An ANOVA was used to compare conidial dimensions of UK and continental Europe isolates with those of NA (geographic location), and among isolates within the two major geographic regions. Geographic region was treated as a fixed effect and isolates as a random effect in the models. Leaves with symptoms when infected with each of the 10 UK and continental Europe isolates and the 10 NA isolates were harvested from the same plants and pressed at the time conidia were washed from the leaves. The pressed leaves were submitted to the WFBI along with agar cultures of each isolate (Table 1). Live cultures of representative isolates were also deposited into the IMI collection (Table 1).

### 2.9 Fungicide sensitivity testing and molecular analyses

Ten isolates of the light leaf spot pathogen, including four reference UK and continental Europe isolates with different sensitivity profiles to carbendazim and prothioconazole, and six NA isolates that had not previously been tested for sensitivity to these fungicides (Tables 1 and 4), were initiated from −80 °C glycerol stocks onto 3% MEA plates. After 3 weeks, 1 ml SDW was added to the colony surface of each isolate and agitated using a sterilized, bent glass rod. Each conidial suspension was filtered through sterilized cheesecloth and adjusted to 10^5^ conidia/ml. A 10 μl droplet of conidial suspension was placed on the centre of a plate of PDA (60 mm diameter × 15 mm deep, with 10 ml medium per plate) containing: (a) no fungicide, (c) 0.39 μg carbendazim/ml, or (c) 1.56 μg prothioconazole/ml. Each isolate was tested on three amended agar plates for each of the three treatments. Plates were dried in a laminar flow hood for 10 min, sealed with a double layer of Parafilm, incubated for 18 days in the dark at 18 °C, and examined for the presence or absence of visible fungal colonies. In addition, the *β‐tubulin* gene sequences from 12 NA isolates (Table 1) were examined for the presence of key amino acid substitutions that have previously been correlated with resistance to MBC fungicides in some isolates from the UK and continental Europe (Carter *et al.*, 2013).

**Table 4:** Discriminatory dose testing of isolates of *Pyrenopeziza* from the United Kingdom, continental Europe, and North America associated with brassica light leaf spot to assess sensitivity to the fungicides carbendazim and prothioconazole

Geographic region (lineage) and isolate code	Geographic origin	Original *Brassica* host	Fungal colonies present or absent on each of three replicate plates^a^		
			No fungicide (control)	Carbendazim (0.39 μg/ml)	Prothioconazole (1.56 μg/ml)
Continental EU and UK (Lineage 1)					
FR2^b^	Le Rheu, France	*B. napus*	+/+/+	−/−/−	−/−/−
UK73^b^	Angus, UK	*B. napus*	+/+/+	+/+/+	P/P/P
8CAB^b^	East Lothian, UK	*B. oleracea*	+/+/+	+/+/+	+/+/+
2016‐50	Northumberland, UK	*B. napus*	+/+/+	+/+/+	P/P/P
North America (Lineage 2)					
Cyc001	Benton Co., OR, USA	*B. rapa*	+/+/+	−/−/−	−/−/−
Cyc011A	Skagit Co., WA, USA	*B. rapa*	+/+/+	−/−/−	−/−/−
Cyc013A	Skagit Co., WA, USA	*B. rapa*	+/+/+	−/−/−	−/−/p
Cyc015	Skagit Co., WA, USA	*B. juncea*	+/+/+	−/−/−	−/−/−
Cyc017	Skagit Co., WA, USA	*B. rapa*	+/+/+	−/−/−	−/−/−
Cyc025	Snohomish Co., WA, USA	*B. rapa*	+/+/+	−/−/−	−/−/−

a Isolates were grown for 18 days in the dark on 3% malt extract agar plates that contained either no fungicide, 0.39 μg carbendazim/ml, or 1.56 μg prothioconazole/ml. Each isolate was tested in triplicate for each treatment. Results were scored as follows: ‘+’ = large colonies visible (>1 cm diameter); ‘−’ = no colony of any size visible; ‘P’ = multiple pinhead colonies (each ≤ 1 mm diameter) visible; ‘p’ = a single pinhead colony (≤1 mm diameter) visible.

b Reference isolates previously characterized as sensitive (FR2), moderately resistant (UK73), or resistant (8CAB) to carbendazim. EC_50_ values for sensitivity of these reference isolates to prothioconazole had previously been determined to be 0.14 (FR2), 1.23 (UK73), and 3.00 (8CAB) μg/ml (Carter *et al.*, 2013).

## 3 RESULTS

### 3.1 Genus confirmation

Phylogenetic analysis of the ITS rDNA of 18 UK, continental European, and OC isolates of *P. brassicae* obtained from *B. napus*, *B. oleracea*, and *B. rapa* plants; 12 NA isolates obtained from *B. juncea*, *B. napus*, *B. rapa*, and *Raphanus* spp.; and 57 isolates of closely related fungi, revealed the NA isolates to group most closely with isolates of *P. brassicae* (Figure 1a). None of the ITS rDNA sequences of the seven other *Pyrenopeziza* species or other closely related fungal genera grouped with the NA isolates. Thus, the NA isolates were confirmed to be a *Pyrenopeziza* sp. most closely related to *P. brassicae*.

### 3.2 Multilocus sequence analysis

Bayesian phylogenetic analyses of the ITS rDNA (Figure 1a), *β‐tubulin* (Figure 1b), and *TEF1‐α* sequences (Figure 1c), as well as the concatenated sequences (Figure 1d) all revealed the UK, continental European, and OC isolates of *P. brassicae* formed a genetically distinct lineage, henceforth referred to as Lineage 1, from the NA isolates, henceforth referred to as Lineage 2. Maximum‐likelihood analyses of the same sequences (ITS rDNA in Figure S1a, *β‐tubulin* in Figure S1b, *TEF1‐α* sequences in Figure S1c, and the concatenated sequences in Figure S1d) gave very similar results. Both Bayesian and maximum‐likelihood analyses supported two distinct lineages that were defined solely by geographic origin, with no evidence for additional grouping based on the *Brassica* or *Raphanus* species from which the isolates originated. These two lineages were more similarly related to each other than to sequences of any other related fungal genera examined for all DNA regions evaluated (Figure 1; Figure S1). The partial ITS rDNA sequence (GenBank accession no. MN028386) obtained from the type herbarium specimen *of P. brassicae* (IMI81823) showed this isolate grouped into Lineage 1.

### 3.3 Mating type screening, distribution, and phylogeny

All of the light leaf spot isolates produced a single amplicon when screened with the multiplex mating type diagnostic PCR assay developed by Foster *et al*. (2002). Lineage 1 isolates produced amplified DNA fragments of either 687 bp for the *MAT1‐1* isolates or 858 bp for the *MAT1‐2* isolates. In contrast, for Lineage 2 isolates, *MAT1‐1* isolates yielded an approximately 786 bp product, which was smaller than the 687 bp product for Lineage 1 isolates, whereas *MAT1‐2* isolates produced an approximately 858 bp fragment of similar size to that of the Lineage 1 isolates. Sequence analyses revealed that the larger product size for *MAT1‐1* in Lineage 2 isolates was due to a 99 bp indel that coded for an additional 33 amino acids targeted by the primers (Singh and Ashby, 1998); no reading frame disruption or premature stop codons were observed in the translated amino acid sequence.

Examination of mating type distributions did not reveal statistically significant deviations from a 1:1 ratio for the 33 Lineage 1 isolates of *P. brassicae* (15:18 *MAT1‐1*:*MAT1‐2* isolates: χ^2^ = 0.273, 1 *df*, *p* = .60) or the 16 Lineage 2 isolates (8:8 *MAT1‐1*:*MAT1‐2* isolates: χ^2^ = 0, 1 *df*, *p* = 1.00). Both *MAT1‐1* and *MAT1‐2* type isolates of Lineage 2 were present in Oregon and Washington. Inspection of sequences of the *MAT1‐1‐3* gene from *MAT1‐1* isolates and *MAT1‐2‐1* gene from *MAT1‐2* isolates also clearly resolved the two lineages, with 90.36% similarity for *MAT1‐1* isolates and 93.24% for *MAT1‐2* isolates (data not shown).

### 3.4 Rep‐PCR DNA fingerprinting

**Figure 3:**
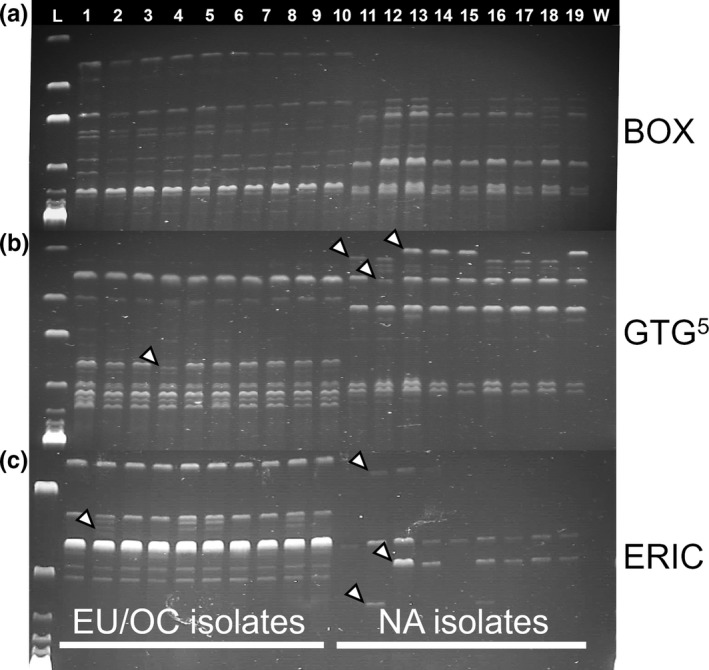
Rep‐PCR DNA fingerprinting of 19 isolates of *Pyrenopeziza brassicae* associated with brassica light leaf spot. Three variants of the rep‐PCR assay were used: (a) BOX PCR, (b) GTG^5^ PCR, and (c) ERIC PCR. The isolates in lanes 1–19 are: PB12, 8CAB, E3A, UK73, a UK field isolate, 17KALE02, 2016‐9, 2016‐34, 2016‐50, CBS157.35, Cyc013A, Cyc015, Cyc017, Cyc025, 14CC2, 14CC4A, 14CC6, 14CC8A, 15LS13B (see Table 1 for isolate details). Geographic origin of the isolates (EU/OC = continental Europe, UK, and Oceania; NA = North America) is noted at the base. Lanes 1–10 = Lineage 1 isolates, lanes 11–19 = Lineage 2 isolates, lane L = Hyperladder 1 (Bioline), and lane W = no‐template water (control) sample. Differences between the two groups of isolates based on DNA fingerprint bands are indicated with white arrowheads

All three rep‐PCR variants tested (BOX, ERIC, and GTG^5^) consistently resolved Lineage 1 isolates of the light leaf spot pathogen from Lineage 2 isolates (Figure 3). Evidence for high genotypic variability was also observed for the ERIC and GTG^5^ data, with unambiguous bands scored as present/absent for each isolate (Figure 3 bands scored with arrows). Based on scoring of bands, 3 of 10 Lineage 1 isolates (30%), and 7 of 9 Lineage 2 isolates (78%) had unique genotypes.

### 3.5 Pathogenicity of Lineage 2 isolates

The 17 isolates from Lineage 2 that were tested for pathogenicity on the turnip (cv. Hakurei) and mustard (cv. Caliente 199) plants all caused chlorotic, rapidly expanding, foliar lesions on both hosts (Figure 2c). Symptoms were not observed on SDW‐treated control plants of either species. Data met assumptions for parametric analysis in pathogenicity tests 1 and 2, but data for pathogenicity test 3 had to be square root‐transformed to meet assumptions of equal variance. Based on the ANOVAs, significant differences in disease severity were detected 21 dai between the turnip and mustard plants (*p* = .0004, *p* < .001, and *p* < .001 for tests 1, 2, and 3, respectively). The turnip plants developed more severe symptoms (100%, 99.7 ± 0.3%, and 84.1 ± 3.8% of the leaf area with symptoms in tests 1, 2, and 3, respectively) than the mustard plants (84.8 ± 3.7%, 77.0 ± 4.0%, and 21.5 ± 2.9% severity, respectively). In addition, turnip plants developed symptoms earlier than mustard plants, with pale brown streaks on the stems and veinal browning on the leaves that darkened over time. Veinal browning was followed by development of small (<5 mm diameter), chlorotic leaf spots, that became diffuse and expanded rapidly, coalescing and covering most of the leaf surface by 21 dai (Figure 2c). Symptoms were similar but developed more slowly on mustard leaves (3–5 days more slowly). Hyaline, smooth, cylindrical, mostly aseptate and eguttulate conidia were observed on short, non‐branching conidiophores in pale acervuli (Figure 2d) on leaves with symptoms from plants inoculated with each of the Lineage 2 isolates. The white, subcuticular conidiomata described by Rawlinson *et al*. (1978) and Fitt *et al*. (1998) as being produced in patches on leaves of plants infected with *P. brassicae* in the UK and continental Europe (Figure 2a,b) were not observed on any of the turnip or mustard plants inoculated with the Lineage 2 isolates. Koch's postulates were completed by reisolating the fungus from leaves with symptoms from all inoculated plants of each species. The fungus could not be reisolated from the control plants of each species. Sequencing the ITS rDNA and *β‐tubulin* regions confirmed that all the reisolates matched the original Lineage 2 isolates (data not shown).

### 3.6 Comparative symptomology caused by isolates of the two lineages

Very different symptoms were observed on turnip plants of the cv. Hakurei inoculated with Lineage 1 isolates compared with those inoculated with Lineage 2 isolate Cyc001. All 10 Lineage 1 isolates produced patches of white conidiomata on leaves, which were first observed 11 dai (Figure 2a,b, photographs taken 14 dai). Patches of white conidiomata were not observed on any of the plants inoculated with the Lineage 2 isolate. Instead, the conidiomata observed were pale tan to brown acervuli and, sometimes, black stromatal knots, that developed when leaves infected with the Lineage 2 isolate were incubated on agar plates or in moist chambers (Figure 2d). By 21 dai, leaves with white conidiomata of the Lineage 1 isolates had senesced more rapidly than plants treated with SDW. The general chlorosis that developed on leaves inoculated with the 10 Lineage 1 isolates differed from the bright yellow chlorotic spots observed on plants inoculated with the Lineage 2 isolate (Figure 2c).

In the first pathogenicity test, there were significant differences among isolates for all three variables measured. For the number of inoculated leaves that turned necrotic, there was a significant main effect of isolates (*p* < .0001). However, there were no significant differences in the mean number of necrotic inoculated leaves caused by 9 of the 10 Lineage 1 isolates and the Lineage 2 isolate, Cyc001, by 28 dai (4.50–5.75 necrotic leaves per plant, *p *> .05; Figure S2a). Only isolate 2016‐5 caused fewer necrotic leaves (4.50 per plant) than that caused by Lineage 2 isolate Cyc001. The control plants averaged 2.50 ± 0.29 necrotic leaves per plant, which was less than that of any of the inoculated plants. In the repeat test, the main effect of isolates was again significant (*p* < .0001). The Lineage 2 isolate Cyc001 caused the greatest number of necrotic leaves (4.00 ± 0.41 per plant), followed by the Lineage 1 isolate 2016‐34 (2.75 ± 0.63 necrotic leaves per plant). Three of the Lineage 1 isolates and the control plants all had <1 necrotic leaf per plant.

The main effect of isolates also significantly affected the number of chlorotic leaves per plant (*p* = .012 in Trial 1). Lineage 2 isolate Cyc001 caused the greatest number of leaves to turn chlorotic by 28 dai (1.8 ± 0.3 and 2.5 ± 0.7 leaves per plant in Trials 1 and 2, respectively; Figure S2b). However, this did not differ significantly from that caused by four Lineage 1 isolates in the first trial and two Lineage 1 isolates in the repeat trial (means separation based on nonparametric rank analyses). All other Lineage 1 isolates caused fewer chlorotic leaves to develop per plant than that caused by Lineage 2 isolate Cyc001 in both trials. None of the control plants developed chlorotic leaves. For the number of leaves with patches of white conidiomata, the negative control plants and plants inoculated with Cyc001 were excluded from the ANOVA, as white conidiomata did not develop on those plants (Figure S2c). Of the 10 Lineage 1 isolates of *P. brassicae* tested, there was a significant effect of isolates (*p* = .005). Isolate 2016‐26 caused the greatest number of leaves to produce patches of white conidiomata (4.25 ± 0.63 leaves per plant), while UK73 caused the fewest leaves to develop white conidiomata (0.50 ± 0.29 leaves per plant). The other isolates did not differ significantly. Very similar results for number of chlorotic leaves per plant and number of leaves with white conidiomata per plant were observed in the repeat trials (data not shown). Koch's postulates were completed by reisolating the fungus (confirmed by sequencing) from foliar lesions of plants inoculated with the Lineage 2 isolate or from white conidiomata that developed on leaves of plants inoculated with the Lineage 1 isolates. Fungi were not reisolated from any of the control plants.

### 3.7 Sexual compatibility testing

In vitro crosses on plates of 3% MEA between Lineage 1 isolates of *P. brassicae* of *MAT1‐1* and *MAT1‐2* types resulted in mature apothecia developing for 22 of the 25 crosses (88%; Table 3). Asci and ascospores were subsequently confirmed in 19 of these 25 crosses (76%) after 9 weeks. By contrast, attempts at inducing sexual reproduction under similar conditions were unsuccessful between Lineage 2 isolates of opposite *MAT1‐1* and *MAT1‐2* types, and between Lineage 1 and Lineage 2 isolates of opposite *MAT* types. Structures that appeared to be apothecial initials were observed in some crosses of Lineage 1 × Lineage 2 isolates but none of these developed into mature apothecia with ascospores (Table 3). Apothecial initials did not develop in any of the attempted *MAT1‐1* and *MAT1‐2* crosses among Lineage 2 isolates.

### 3.8 Morphological analysis

**Figure 4:**
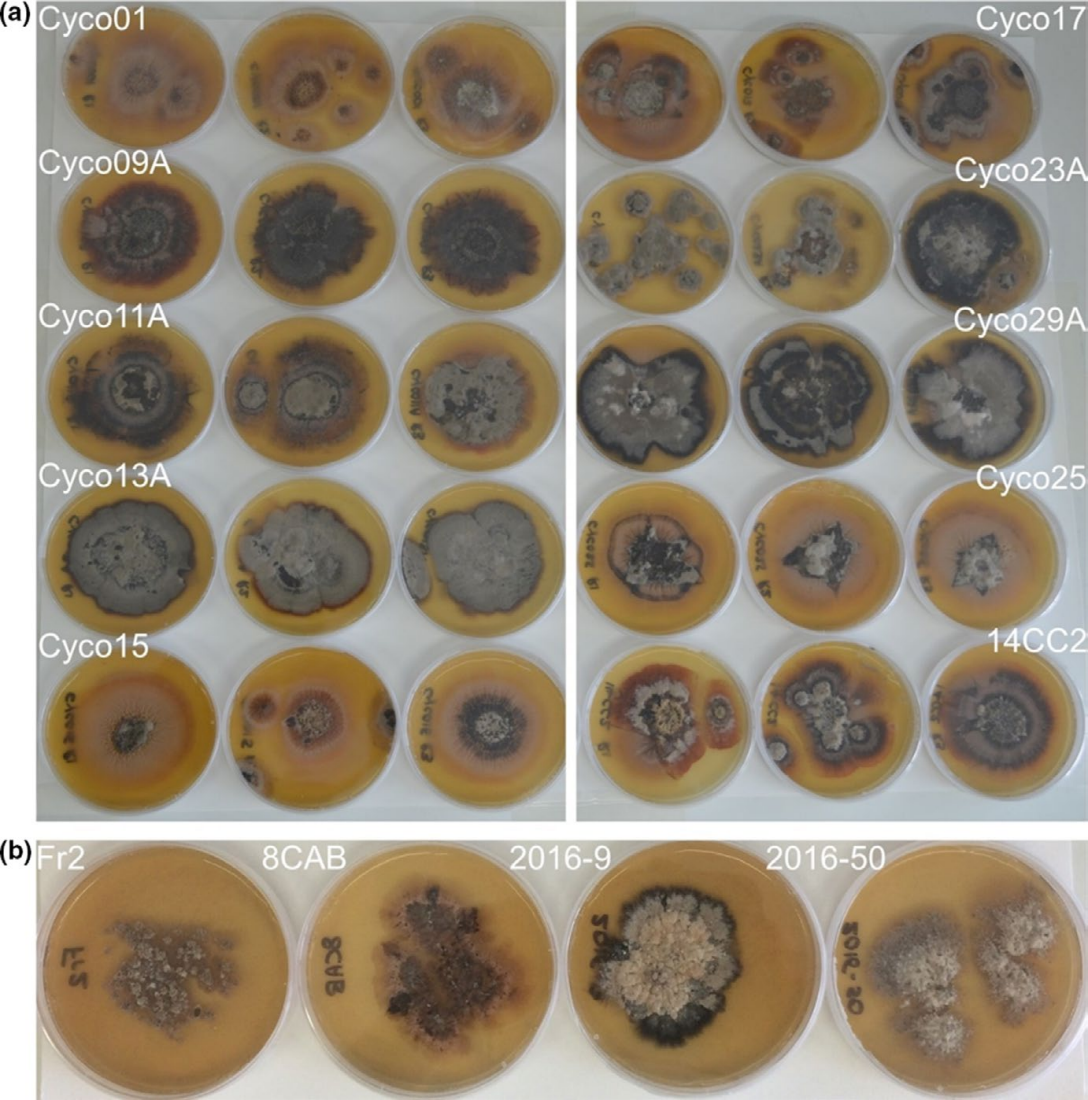
Variation in colony morphology of isolates of *Pyrenopeziza brassicae* associated with brassica light leaf spot that were grown on 3% malt extract agar for 4 months. (a) Ten North American (NA) isolates of Lineage 2 (three replicates of each shown); note the phenotypic variation among isolates, which was consistent among replicate plates with the exception of Cyc023A. (b) Four United Kingdom and continental European isolates of Lineage 1 of *P. brassicae* showing overlapping colony morphology with that of NA isolates. Isolates from NA, the UK, and continental Europe (EU) could not be distinguished based on colony appearance

Considerable colony variation was evident among the 10 Lineage 2 isolates of the light leaf spot pathogen, with diverse pigment colours (black, brown, grey, pink, red, and yellow; Figure 4a). For all Lineage 2 isolates examined (except Cyc023A), the observed phenotype was consistent among the three replicate cultures on MEA. Additional comparisons of the 10 Lineage 2 isolates with four representative Lineage 1 isolates revealed no obvious differences in colony phenotype that distinguished isolates from the two major geographic regions (Figure 4a,b).

Examination of conidia produced in vitro by colonies growing on 3% MEA for 6 weeks revealed it was not possible to distinguish between the 10 Lineage 1 and 8 Lineage 2 isolates based on shape of the conidia. All 18 isolates produced hyaline, usually aseptate, and cylindrical conidia. Moreover, there was no significant difference among the Lineage 1 versus Lineage 2 isolates for conidial length (Lineage 1 isolates averaged 8 ± 0.13 µm [mean ± *SE*] for 250 conidia, and Lineage 2 isolates averaged 7.80 ± 0.12 µm for 200 conidia; Student's *t* test = 1.23, *df* = 448, *p* = .262) or diameter (Lineage 1 isolates averaged 2.23 ± 0.03 µm for 250 conidia, and Lineage 2 isolates averaged 2.18 ± 0.03 µm for 200 conidia; Student's *t* test = 1.11, *df* = 448, *p* = .268).

In contrast, when conidia were washed directly from leaves of the turnip cv. Hakurei with symptoms, 28 dai of the plants with 10 Lineage 1 isolates and 10 Lineage 2 isolates, significant differences were observed in morphology of conidia produced by isolates from the two major geographic regions. A single septum was observed in some conidia collected from leaves inoculated with most (9 of 10) Lineage 2 isolates but only from leaves inoculated with 1 of the 10 Lineage 1 isolates. The number of conidia with a septum averaged 5.3 ± 1.1 for 60 conidia measured per isolate for the 10 Lineage 2 isolates compared to 0.1 ± 0.1 for 60 conidia per isolate for the Lineage 1 isolates (*p* < .0001). Conidial width did not differ significantly (*p* = .1300, *R* ^2^ = .39) among all 20 isolates, but was significantly greater for the 10 Lineage 1 isolates (average of 4.41 ± 0.02 µm) than for the 10 Lineage 2 isolates (3.14 ± 0.17 µm; *p* < .0001, *R* ^2^ = .60). Conidial length differed significantly among the 20 isolates (*p* = .0135, *R* ^2^ = .47), and between the 10 Lineage 1 isolates compared to the 10 Lineage 2 isolates (*p* < .0001, *R* ^2^ = .60, respectively). Conidial length averaged 10.08 ± 0.07 µm for the 10 Lineage 2 isolates versus 11.70 ± 0.06 µm for the 10 Lineage 1 isolates. In summary, the 10 Lineage 2 isolates produced slightly shorter and narrower conidia in planta than the 10 Lineage 1 isolates, and 90% of the Lineage 2 isolates produced a few septate conidia in planta, whereas only one of the 10 Lineage 1 isolates formed septate conidia in planta.

### 3.9 Fungicide sensitivity testing and molecular analysis

In vitro testing showed the six Lineage 2 isolates to be very sensitive to carbendazim, as no fungal growth was observed on any of the agar plates amended with 0.39 μg/ml carbendazim (Table 4). This contrasted with Lineage 1 isolates of *P. brassicae* known to be moderately and highly resistant to carbendazim, UK73 and 8CAB, respectively. Subsequent inspection of the β‐tubulin amino acid sequences from 12 Lineage 2 isolates revealed none contained the E198A, E198G, F220Y, or L240F substitutions that have been associated with MBC resistance in some UK *P. brassicae* isolates (Carter *et al.*, 2013). Additional sensitivity testing revealed the six Lineage 2 isolates to be sensitive to prothioconazole, as no fungal growth was observed on agar medium amended with 1.56 μg/ml, with the exception of one replicate plate of Lineage 2 isolate Cyc013A, on which a single colony <1 mm in diameter was observed. This contrasted with the growth observed for UK isolates UK73 and 8CAB, for which EC_50_ values had previously been determined to be ≥1.23 μg/ml (Carter *et al.*, 2014).

## 4 DISCUSSION

In this study, isolates of the light leaf spot pathogen from three major geographic regions were resolved into two closely related but genetically distinct phylogenetic lineages. The first (Lineage 1) contained isolates from the UK, continental Europe, and OC that originated from *B. napus*, *B. oleracea*, and *B. rapa* plants, and included the type specimen of *P. brassicae*, IMI81823 (Rawlinson *et al.*, 1978) for which only a partial ITS rDNA sequence could be generated from the herbarium specimen. The second (Lineage 2) included NA isolates that originated from *B. juncea*, *B. napus*, *B. rapa*, and *Raphanus* spp. from western Oregon and western Washington. The two lineages were distinguished consistently based on: (a) Bayesian and maximum‐likelihood analyses of individual sequences and MLSA of concatenated sequences of the ITS rDNA as well as the *β‐tubulin* and *TEF1‐α* genes; (b) phylogenetic analyses of *MAT1‐1* and *MAT1‐2* sequences; and (c) rep‐PCR DNA fingerprinting (including BOX, ERIC, and GTG^5^ variants). In addition, *MAT1‐1* type Lineage 2 isolates contained a 99 bp indel in the *MAT1‐1‐3* gene that was not present in any of the Lineage 1 isolates of *P. brassicae* examined. The two lineages were discriminated exclusively based on geographic origin, with no additional subdivision based on original host species.

Pathogenicity tests in greenhouse and growth chamber conditions revealed strikingly different foliar symptoms on *B. rapa* seedlings inoculated with Lineage 1 versus Lineage 2 isolates. All 10 Lineage 2 isolates caused bright yellow chlorotic spots, each of which developed a necrotic centre and veinal browning. These yellow spots expanded rapidly, remaining chlorotic and leading to leaf chlorosis and eventual necrosis of entire inoculated leaves. Pale tan to light brown acervuli formed in the chlorotic and necrotic leaf tissue, in which conidia were observed when examined microscopically. In contrast, the 10 Lineage 1 isolates resulted in formation of white conidiomata on otherwise “healthy” green leaves, followed by rapid leaf necrosis (sometimes with leaf distortion and crinkling, but never with bright yellow chlorotic lesions). Overall, these results are consistent with the different symptoms observed on naturally infected plants under field conditions on the continents from which the original fungal isolates were obtained (Carmody, 2017; Karandeni Dewage *et al.*, 2018).

Isolates of *MAT1‐1* and *MAT1‐2* types were found for both Lineage 1 and Lineage 2. In vitro crosses between Lineage 1 isolates of MAT1‐1 and MAT1‐2 types resulted in development of mature apothecia with asci and ascospores for a majority of the crosses (76%) within 9 weeks of pairing the isolates, which is consistent with previous studies (Ilott *et al.*, 1984). Conversely, mature sexual structures were not observed in similar crosses between Lineage 2 isolates of opposite *MAT* type, i.e., no sexual cycle could be confirmed. A few of the attempted sexual crosses between Lineage 1 and Lineage 2 isolates of opposite *MAT* type did result in what appeared to be apothecial initials, but these structures did not develop into mature apothecia with asci and ascospores. One possibility is that the apothecial initials observed in these interlineage crosses could have resulted solely from the Lineage 1 isolate, as Ilott *et al*. (1984) reported that some UK isolates produced what appeared to be apothecial initials even in single‐isolate cultures. The inability to confirm sexual reproduction between the two lineages of opposite mating type might be explained by the sequence divergence observed at the *MAT1‐1* locus, i.e., the 99 bp indel detected in the *MAT1‐1* Lineage 2 isolates but not in Lineage 1 isolates of this mating type. Further work is needed to investigate the possibility of sexual compatibility between isolates of Lineages 1 and 2, and the results of this study should be interpreted with caution given the limited number of isolates tested and the limited conditions under which the isolates were tested for sexual compatibility. It is possible that Lineage 2 isolates may have different in vitro development requirements for induction of a sexual cycle, given that no sexual stage has yet been identified in the Pacific Northwest region of the USA, where this pathogen was first detected in NA.

The Lineage 2 isolates of the light leaf spot pathogen exhibited several “signatures of sexuality” that are indicative of cryptic sexual potential. First, the ratio of *MAT1‐1*:*MAT1‐2* type isolates did not deviate significantly from a 1:1 distribution, as is typically the case under frequency‐dependent selection operating on *MAT* genes (Milgroom, 1996). Secondly, the Lineage 2 isolates exhibited high genotypic (based on rep‐PCR DNA fingerprinting) and phenotypic (based on colony morphology on 3% MEA) diversity, as is usually observed with sexually outcrossing populations (McDonald and Linde, 2002). The Lineage 2 isolates appeared more diverse (seven of nine isolates had a unique rep‐PCR genotype) than the Lineage 1 isolates (3 of 10 isolates had a unique genotype). Further work is required to investigate possible cryptic sexuality in Lineage 2 isolates, including more extensive attempts at sexual crossing, e.g., in planta on senescing host debris (Gilles *et al.*, 2001). The presence of a sexual cycle in Lineage 2 could affect pathogen dispersal and, potentially, increase the risk of breakdown in effectiveness of some disease management strategies, e.g., from development of fungicide resistance and/or the presence of virulence genes in the pathogen population that overcome host plant resistance (McDonald and Linde, 2002).

Morphologically, it was possible to distinguish between conidia of Lineage 1 and 2 isolates produced on infected *B. rapa* plants. Lineage 2 isolates produced slightly shorter and narrower conidia (10.08 ± 0.07 [mean ± *SD*] × 3.14 ± 0.17 µm) than Lineage 1 isolates (11.70 ± 0.06 × 4.41 ± 0.02 µm). In addition, a limited number of conidia produced by Lineage 2 isolates formed a single septum as the conidia aged, whereas only a single isolate of Lineage 1 (of the 10 examined) occasionally produced conidia that developed a septum. By contrast, no differences in conidial dimensions or colony colour were observed between the Lineage 1 and 2 isolates when grown on 3% MEA. Isolates from both lineages formed a range of black, brown, grey, pink, or yellow pigmentation on this medium. The difference in spore dimensions observed for spores of Lineages 1 and 2 generated in vitro versus in vivo could reflect the well‐documented potential impact of substrate (3% MEA vs. live plants in this case) on spore production by many fungi. However, the measurement of spores produced in vitro was done at Rothamsted Research whereas the measurement of spores produced in vivo was done at WSU, which confounded any potential effects of the location and method with differences in spore dimensions among isolates. Given these difficulties with morphological discrimination in vitro between isolates of the two lineages, specific PCR assays have since been designed by King and West at Rothamsted to enable rapid lineage discrimination (data not shown). Such PCR assays could be used to differentiate isolates of the two lineages, including isolates of the two lineages present in infected leaves and seed.

The first report of light leaf spot in NA was in Oregon in 2014, with subsequent widespread distribution of the disease discovered across western Oregon and, more recently, in three counties in Washington State, which suggests fairly rapid spread of the causal agent within the Pacific Northwest USA. Indeed, based on the Lineage 2 isolates evaluated in this study, the pathogen was confirmed as far north as Whatcom Co., WA and as far south as Douglas Co., OR. The geographic origin of Lineage 2 isolates in the USA remains unclear. However, based on this study, Lineage 2 isolates appear not to have originated from the UK, continental Europe, or OC, as isolates from those regions were in the genetically distinct Lineage 1. One possible source of Lineage 2 isolates is Asia. Light leaf spot outbreaks have been reported in Japan and Thailand (Rawlinson *et al.*, 1978; CABI, 2015). Future work to characterize Asian isolates should provide insight on a more global scale of the potential origin of the NA isolates.

Currently, the two lineages appear to be restricted geographically to either the UK, continental Europe, and OC (Lineage 1) or to NA (Lineage 2). Therefore, appropriate precautions are needed to prevent movement of isolates from the different lineages between regions and to other parts of the world. This includes transfer of potentially infected plants or seed (Carmody and du Toit, 2017) on which the pathogen might be present, either with or without symptoms. More comprehensive testing of the responses of *B. napus*, *B. oleracea*, *B. rapa*, and other Brassicaceae germplasm to isolates from the two lineages is needed to assess potential differences in susceptibility of plant germplasm (Boys *et al.*, 2012). Although this study indicated that isolates from Lineages 1 and 2 are sexually incompatible, there remains a risk of hybridization or somatic recombination between isolates of the two groups. Given the recent rapid spread of Lineage 2 across western Oregon and western Washington, there is also a risk of spread into Canada, the world's third largest producer of canola (*B. napus*), and other regions of the USA, as well as Mexico.

Management of light leaf spot in the UK and continental Europe is based primarily on timely applications of efficacious fungicides. Prior to this study, data were not available on the sensitivity of Lineage 2 isolates of the light leaf spot pathogen to fungicides used to control this disease in the UK and continental Europe. Phenotypic screening of six Lineage 2 isolates revealed all to be sensitive to both carbendazim and prothioconazole. Examination of the β‐tubulin amino acid sequences of Lineage 2 isolates revealed 100% identity to that of a UK isolate previously classified as sensitive to MBC fungicides (KC342227; Carter *et al.*, 2013), with no evidence for the key substitutions (e.g., E198A or L240F) that have been correlated with MBC resistance in Lineage 1 isolates (Carter *et al.*, 2013). Although more isolates should be tested, it appears likely that Lineage 2 isolates might be controlled effectively with applications of MBC and DMI fungicides, as demonstrated recently with MBC and DMI fungicide seed treatments evaluated with a mustard seed lot infected with a Lineage 2 isolate (Carmody and du Toit, 2017). However, given the emergence of resistance to both fungicide groups in some Lineage 1 isolates (Carter *et al.*, 2013, 2014), implementation of fungicide resistance management strategies by NA brassica growers will be important to extend the effective life of these fungicides against the pathogen (e.g., using mixtures or rotations of fungicides with different modes of action).

In conclusion, based on the CSC that combines morphological, ecological, biological, and genetic (phylogenetic) data (Crous *et al.*, 2015), convincing evidence was generated in this study for two genetically distinct evolutionary lineages of *P. brassicae*, with Lineage 1 comprising isolates from the UK, continental Europe, and OC, including the type specimen, IMI81823 (Rawlinson *et al.*, 1978); and Lineage 2 comprising NA isolates. More detailed morphological, genetic, and biological assessment of a broader collection of isolates from additional geographic locations and other *Pyrenopeziza* species should enable determination of whether the NA isolates represent a new species. Furthermore, given distinct differences in symptoms and signs (types of conidiomata) observed on *B. rapa* and *B. juncea* plants inoculated with isolates of the two lineages, and symptoms observed on both inoculated and naturally infected plants of *B. juncea*, *B. napus*, *B. oleracea*, *B. rapa*, and *Raphanus sativus* (Claassen, 2016; Carmody, 2017), we propose the common name “chlorotic leaf spot” be used to describe the disease caused by Lineage 2 isolates in order to differentiate this disease from classic light leaf spot symptoms caused by isolates of Lineage 1 of *P. brassicae*.

## CONFLICTS OF INTEREST

The authors have no conflicts of interest to declare.

## Data Availability

The data that support the findings of this study are available from the corresponding author upon reasonable request.

## References

[ppa13137-bib-0001] Anderson, P.K. , Cunningham, A.A. , Patel, N.G. , Morales, F.J. , Epstein, P.R. and Daszak, P. (2004) Emerging infectious diseases of plants: pathogen pollution, climate change and agrotechnology drivers. Trends in Ecology & Evolution, 19, 535–544. 16701319 10.1016/j.tree.2004.07.021

[ppa13137-bib-0002] Bakkeren, G. , Kronstad, J.W. and Levesque, C.A. (2000) Comparison of AFLP fingerprints and ITS sequences as phylogenetic markers in Ustilagomycetes. Mycologia, 92, 510–521.

[ppa13137-bib-0003] Boys, E.F. , Roques, S.E. , Ashby, A.M. , Evans, N. , Latunde‐Dada, A.O. , Thomas, J.E. *et al* (2007) Resistance to infection by stealth: *Brassica napus* (winter oilseed rape) and *Pyrenopeziza brassicae* (light leaf spot). European Journal of Plant Pathology, 118, 307–321.

[ppa13137-bib-0004] Boys, E.F. , Roques, S.E. , West, J.S. , Werner, C.P. , King, G.J. , Dyer, P.S. *et al* (2012) Effects of R gene‐mediated resistance in *Brassica napus* (oilseed rape) on asexual and sexual sporulation of *Pyrenopeziza brassicae* (light leaf spot). Plant Pathology, 61, 543–554.

[ppa13137-bib-0005] Carmody, S.M. (2017) Light leaf spot and white leaf spot of Brassicaceae in Washington State. MS thesis, Washington State University.

[ppa13137-bib-0006] Carmody, S.M. and du Toit, L.J. (2017) Seed treatments to eradicate *Pyrenopeziza brassicae* from infected mustard (*Brassica juncea*) seed. Phytopathology, 107, S5.76.

[ppa13137-bib-0007] Carmody, S.M. , Ocamb, C.M. and du Toit, L.J. (2016) Potential seed transmission of *Pyrenopeziza brassicae* and *Mycosphaerella capsellae* in brassicas in the Pacific Northwest USA. Phytopathology, 106, S4.196.

[ppa13137-bib-0008] Carter, H.E. , Cools, H.J. , West, J.S. , Shaw, M.W. and Fraaije, B.A. (2013) Detection and molecular characterisation of *Pyrenopeziza brassicae* isolates resistant to methyl benzimidazole carbamates. Pest Management Science, 69, 1040–1048. 23760810 10.1002/ps.3585

[ppa13137-bib-0009] Carter, H.E. , Fraaije, B.A. , West, J.S. , Kelly, S.L. , Mehl, A. , Shaw, M.W. *et al* (2014) Alterations in the predicted regulatory and coding regions of the sterol 14α‐demethylase gene (*CYP51*) confer decreased azole sensitivity in the oilseed rape pathogen *Pyrenopeziza brassicae* . Molecular Plant Pathology, 15, 513–522. 24298976 10.1111/mpp.12106 PMC6638911

[ppa13137-bib-0010] Centre for Agriculture and Biosciences International . (2015) *Pyrenopeziza brassicae*. Crop Protection Compendium. Wallingford, UK Available at: http://www.cabi.org/cpc/datasheet/46123 [Accessed 5 January 2017].

[ppa13137-bib-0011] Cheah, H. , Hartill, W.T.F. and Corbin, B.J. (1982) Ascospore release in *Pyrenopeziza brassicae* . Transactions of the British Mycological Society, 79, 536–539.

[ppa13137-bib-0012] Claassen, B.J. (2016) Investigations of black leg and light leaf spot on Brassicaceae hosts in Oregon. MS thesis, Corvallis, OR, Oregon State University.

[ppa13137-bib-0013] Crous, P.W. , Groenewald, J.Z. and Gams, W. (2003) Eyespot of cereals revisited: ITS phylogeny reveals new species relationships. European Journal of Plant Pathology, 109, 841–850.

[ppa13137-bib-0014] Crous, P.W. , Hawksworth, D.L. and Wingfield, M.J. (2015) Identifying and naming plant‐pathogenic fungi: past, present, and future. Annual Review of Phytopathology, 53, 247–267. 10.1146/annurev-phyto-080614-120245 26047568

[ppa13137-bib-0015] Darriba, D. , Taboada, G.L. , Doallo, R. and Posada, D. (2012) jModelTest 2: more models, new heuristics and parallel computing. Nature Methods, 9, 772. 10.1038/nmeth.2109 PMC4594756 22847109

[ppa13137-bib-0016] Einax, E. and Voigt, K. (2003) Oligonucleotide primers for the universal amplification of β‐tubulin genes facilitate phylogenetic analyses in the regnum Fungi. Organism Diversity and Evolution, 3, 185–194.

[ppa13137-bib-0017] Fitt, B.D.L. , Doughty, K.J. , Gladders, P. , Steed, J.M. and Sutherland, K.G. (1998) Diagnosis of light leaf spot (*Pyrenopeziza brassicae*) on winter oilseed rape (*Brassica napus*) in the UK. Annals of Applied Biology, 133, 155–166.

[ppa13137-bib-0018] Foster, S.J. , Ashby, A.M. and Fitt, B.D.L. (2002) Improved PCR‐based assays for pre‐symptomatic diagnosis of light leaf spot and determination of mating type of *Pyrenopeziza brassicae* on winter oilseed rape. European Journal of Plant Pathology, 108, 379–383.

[ppa13137-bib-0019] Gilles, T. , Fitt, B.D.L. and Jeger, M.J. (2001) Effects of environmental factors on development of *Pyrenopeziza brassicae* (light leaf spot) apothecia on oilseed rape debris. Phytopathology, 91, 392–398. 18943852 10.1094/PHYTO.2001.91.4.392

[ppa13137-bib-0020] Ilott, T.W. , Ingram, D.S. and Rawlinson, C.J. (1984) Heterothallism in *Pyrenopeziza brassicae*, cause of light leaf spot of brassicas. Transactions of the British Mycological Society, 82, 477–483.

[ppa13137-bib-0021] Inglis, D.A. , du Toit, L.J. and Miller, T.W. (2013) Production of Brassica Seed Crops in Washington State: A Case Study on the Complexities of Coexistence. Pullman, WA: Washington State University Extension Manual EM062E.

[ppa13137-bib-0022] Karandeni Dewage, C.S. , Klöppel, C.A. , Stotz, H.U. and Fitt, B.D.L. (2018) Host–pathogen interactions in relation to management of light leaf spot disease (caused by *Pyrenopeziza brassicae*) on *Brassica* species. Crop and Pasture Science, 69, 9–19.

[ppa13137-bib-0023] Karolewski, Z. , Kaczmarek, J. , Jedryczka, M. , Cools, H.J. , Fraaije, B.A. , Lucas, J.A. *et al* (2012) Detection and quantification of airborne inoculum of *Pyrenopeziza brassicae* in Polish and UK winter oilseed rape crops by real‐time PCR assays. Grana, 51, 270–279.

[ppa13137-bib-0024] Kumar, S. , Stecher, G. and Tamura, K. (2016) MEGA7: Molecular evolutionary genetics analysis version 7.0 for bigger datasets. Molecular Biology and Evolution, 33, 1870–1874. 27004904 10.1093/molbev/msw054 PMC8210823

[ppa13137-bib-0025] Lacey, M.E. , Rawlinson, C.J. and McCartney, H.A. (1987) First record of the natural occurrence in England of the teleomorph of *Pyrenopeziza brassicae* on oilseed rape. Transactions of the British Mycological Society, 89, 135–140.

[ppa13137-bib-0026] Majer, D. , Lewis, B.G. and Mithen, R. (1998) Genetic variation among field isolates of *Pyrenopeziza brassicae* . Plant Pathology, 47, 22–28.

[ppa13137-bib-0027] McDonald, B.A. and Linde, C. (2002) Pathogen population genetics, evolutionary potential, and durable resistance. Annual Review of Phytopathology, 40, 349–379. 10.1146/annurev.phyto.40.120501.101443 12147764

[ppa13137-bib-0028] Milgroom, M. (1996) Recombination and the multilocus structure of fungal populations. Annual Review of Phytopathology, 34, 457–477. 10.1146/annurev.phyto.34.1.457 15012552

[ppa13137-bib-0029] Ocamb, C.M. (2014) Disease Alert – Light Leaf Spot in Crucifer Seed Fields in the Willamette Valley. Corvallis, OR: Oregon State University Available at: http://mtvernon.wsu.edu/path_team/Light%2520leaf%2520spot%2520OSU%2520Disease%2520Alert%2520in%2520crucifers%252017%2520June.pdf [Accessed 5 January 2017].

[ppa13137-bib-0030] Ocamb, C.M. , Mallory‐Smith, C. , Thomas, W.J. , Serdani, M. and Putnam, M.L. (2015) New and re‐emerging fungal pathogens affecting Brassicaceae plants in western Oregon: black leg, light leaf spot, and white leaf spot. Phytopathology, 105, 542‐P.25317843

[ppa13137-bib-0031] Phytosanitary Alert System . (2015) *Pyrenopeziza brassicae* (light leaf spot fungus) – confirmed in Oregon. North American Plant Protection Organization Phytosanitary Alert System. Available at: https://www.pestalerts.org/official-pest-report/pyrenopeziza-brassicae-light-leaf-spot-fungus-confirmed-oregon [Accessed 20 December 2019].

[ppa13137-bib-0032] Rawlinson, C.J. , Sutton, B.C. and Muthyalu, G. (1978) Taxonomy and biology of *Pyrenopeziza brassicae* sp. nov. (*Cylindrosporium concentricum*), a pathogen of winter oilseed rape (*Brassica napus* spp. *oleifera*). Transactions of the British Mycological Society, 71, 425–439.

[ppa13137-bib-0033] Singh, G. and Ashby, A.M. (1998) Cloning of the mating type loci from *Pyrenopeziza brassicae* reveals the presence of a novel mating type gene within a discomycete *MAT1‐2* locus encoding a putative metallothionein‐like protein. Molecular Microbiology, 30, 799–806. 10094628 10.1046/j.1365-2958.1998.01112.x

[ppa13137-bib-0034] Taşkin, H. , Büyükalaca, S. , Doğan, H.H. , Rehner, S.A. and O'Donnell, K. (2010) A multigene molecular phylogenetic assessment of true morels (*Morchella*) in Turkey. Fungal Genetics and Biology, 47, 672–682. 20580850 10.1016/j.fgb.2010.05.004

[ppa13137-bib-0035] Versalovic, J. , Schneider, M. , de Bruijn, F.J. and Lupski, J.R. (1994) Genomic fingerprinting of bacteria using repetitive sequence‐based polymerase chain reaction. Methods in Molecular and Cellular Biology, 5, 25–40.

