# Supplementary figures and images for: A phylogenetically distinct lineage of Pyrenopeziza brassicae associated with chlorotic leaf spot of Brassicaceae in North America
Source: Plant Pathol. 2020 Jan 22;69(3):518–37. doi: 10.1111/ppa.13137 (PMC7074063; doi:10.1111/ppa.13137)

**a**

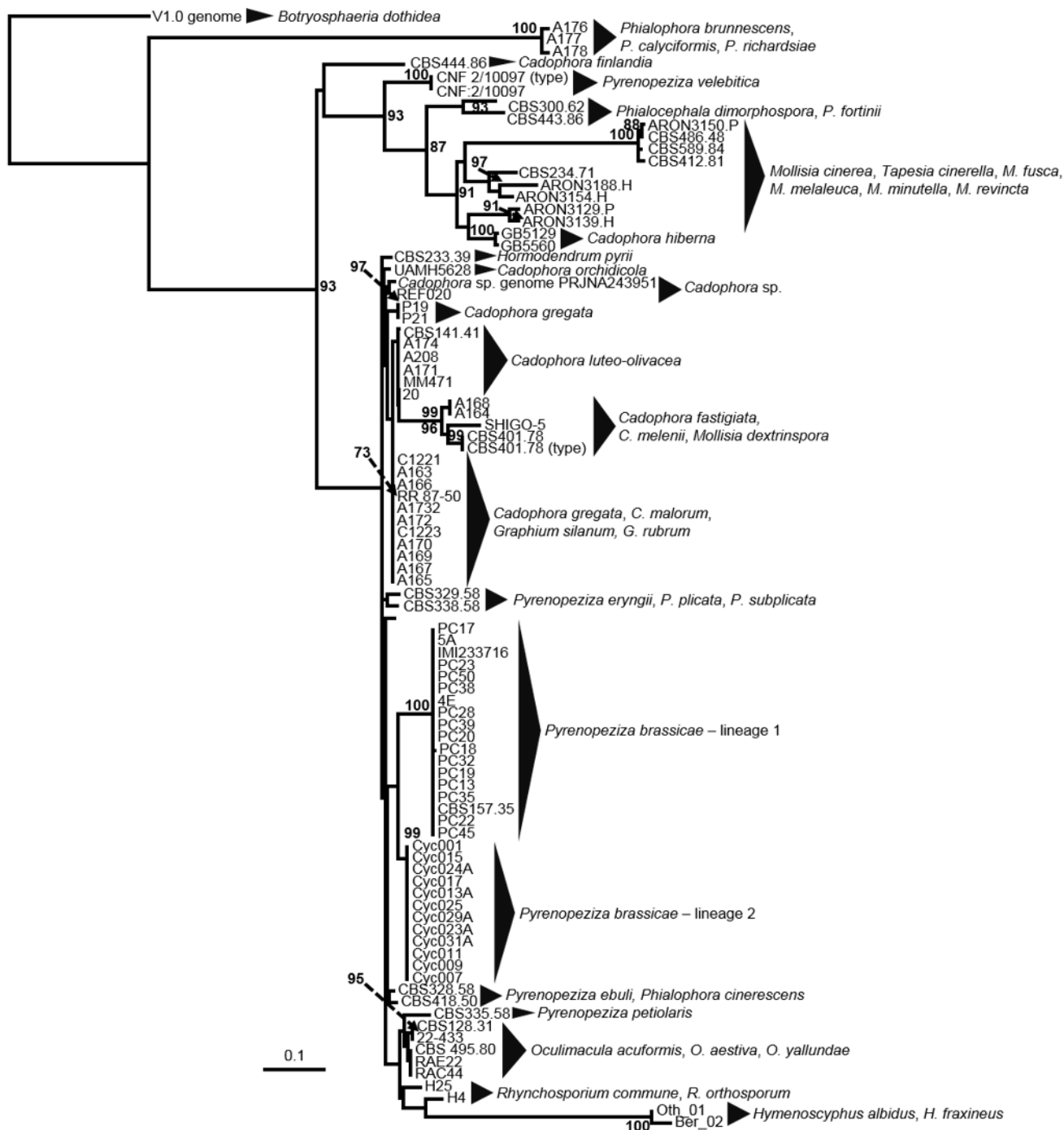

**b**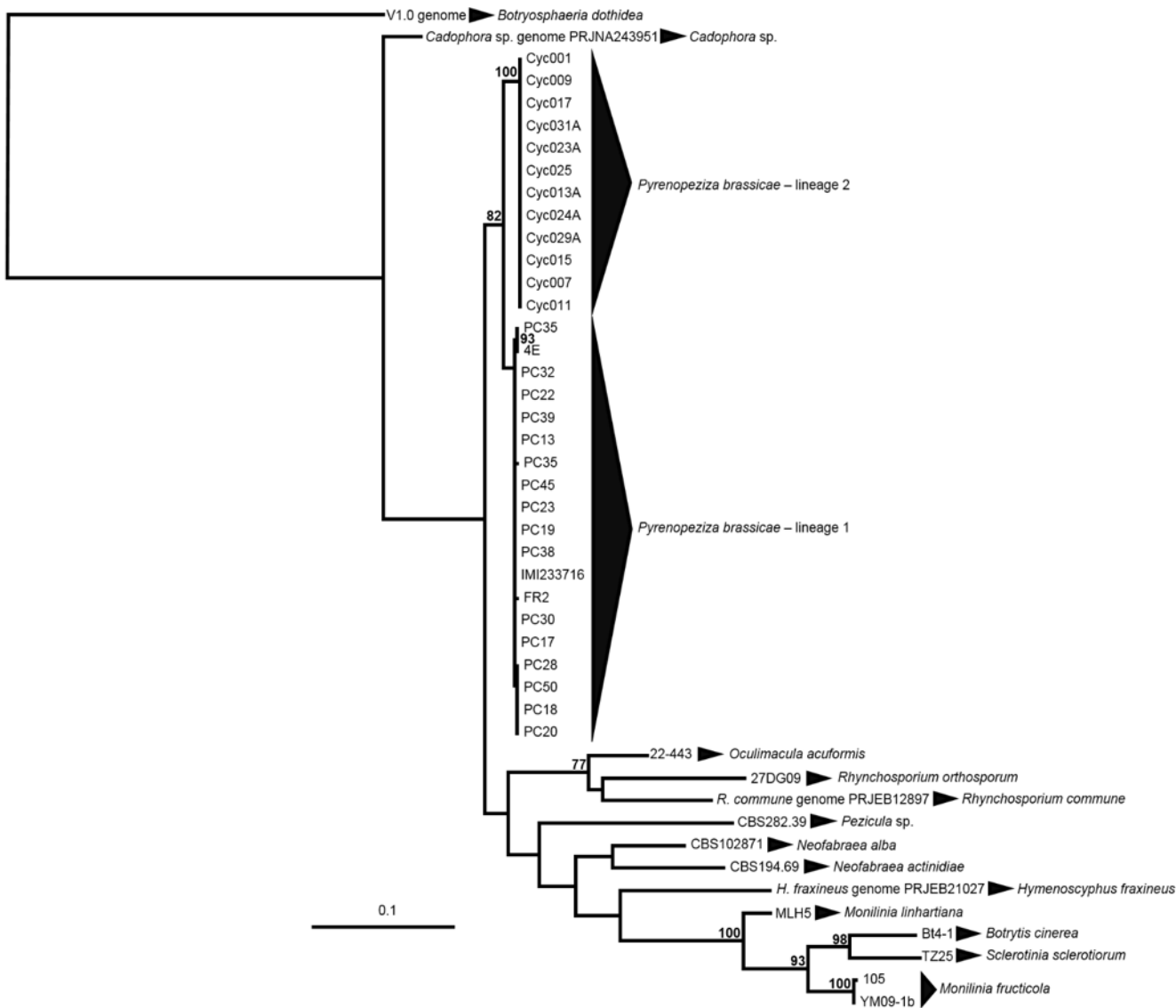

**C**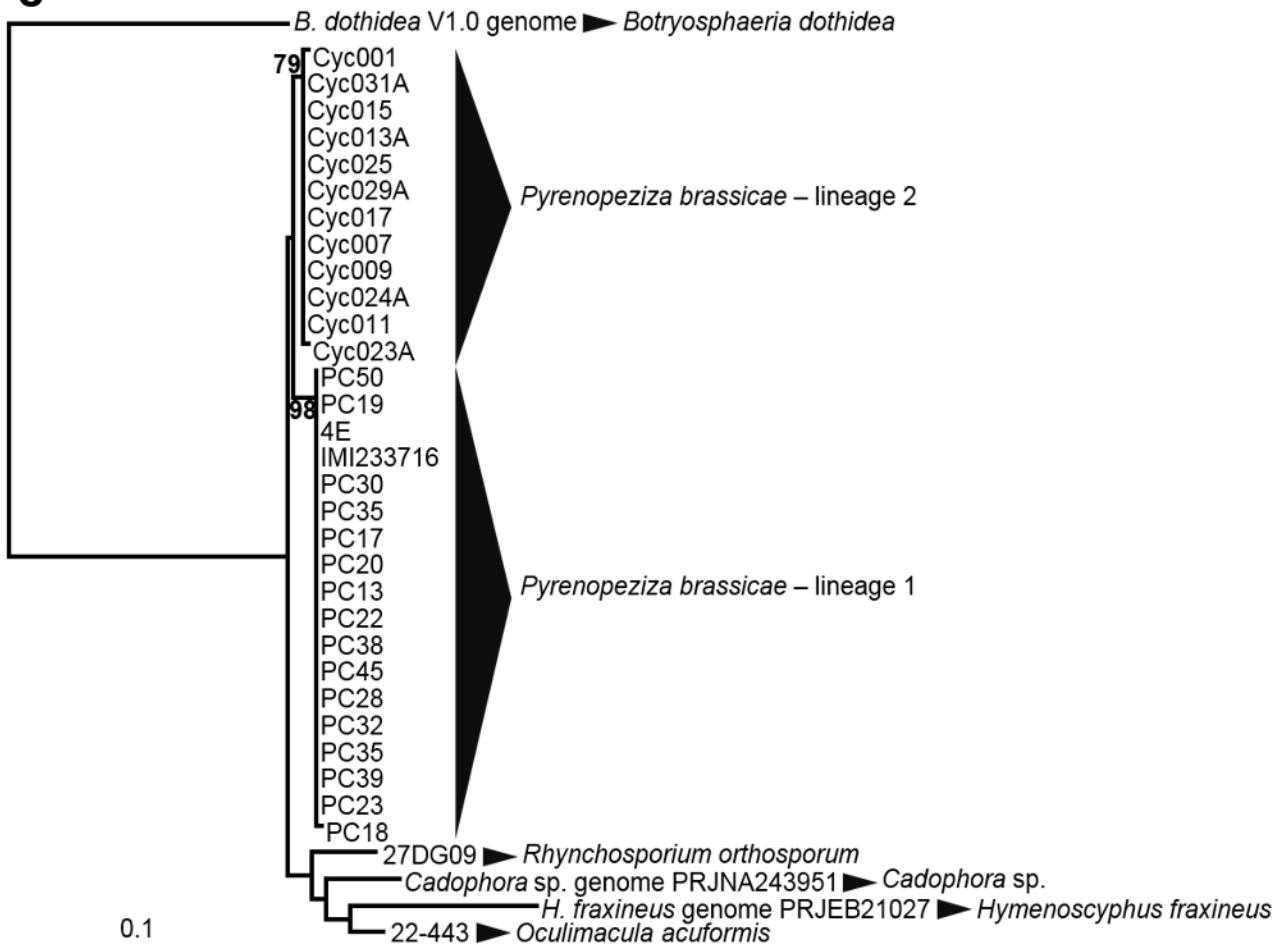

**d**

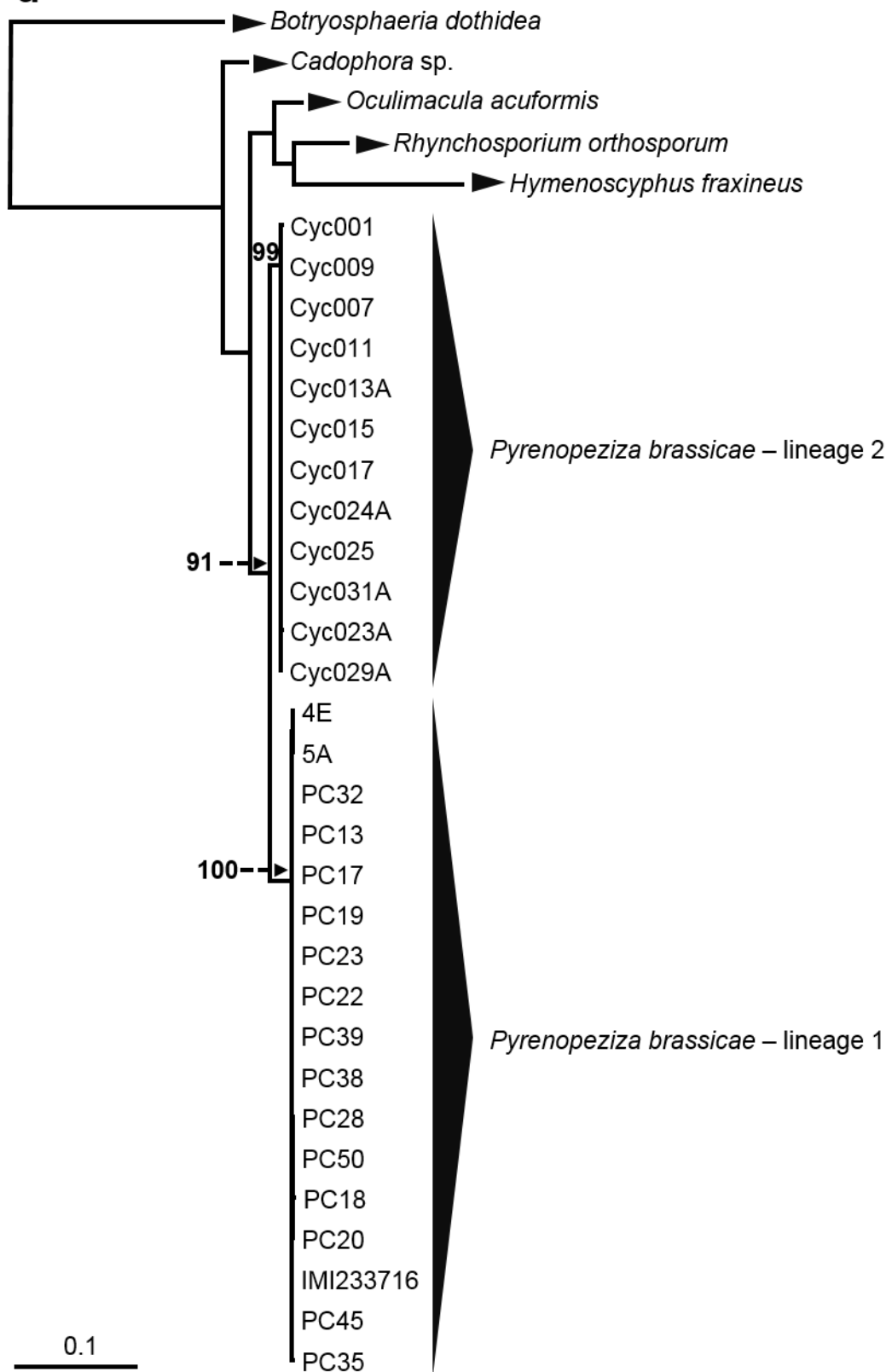

Supplement: Supplementary file 1 [file PPA-69-518-s001.pdf]

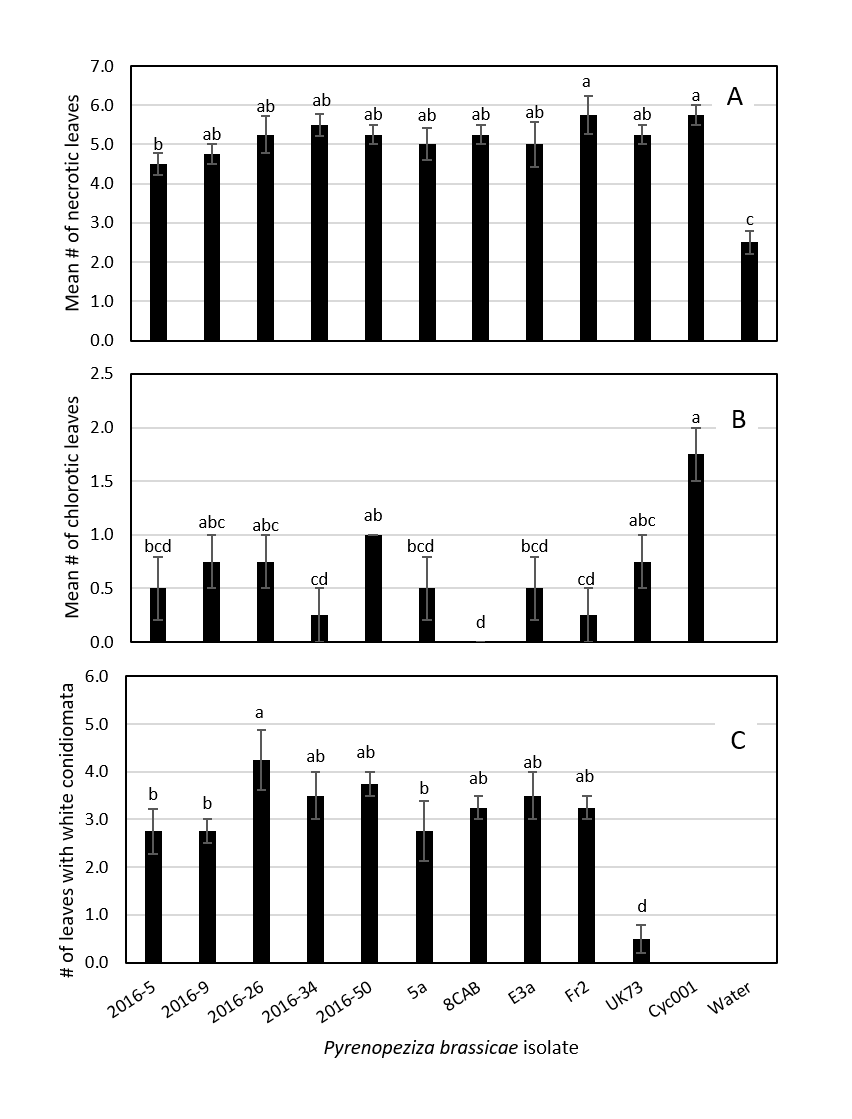

Supplement: Supplementary file 2 [file PPA-69-518-s002.tif]
