# Supplementary material for: A phylogenetically distinct lineage of Pyrenopeziza brassicae associated with chlorotic leaf spot of Brassicaceae in North America
Source: Plant Pathol. 2020 Jan 22;69(3):518–37. doi: 10.1111/ppa.13137 (PMC7074063; doi:10.1111/ppa.13137)
Supplement: Supplementary file 3 [file PPA-69-518-s003.docx]

SUPPLEMENTARY TABLE 1. GenBank accession numbers for the internal transcribed spacer (ITS) region of ribosomal DNA (rDNA), *beta tubulin* (*β-tubulin*) gene, and *translation elongation factor 1-alpha* (*TEF1-α*) gene sequences used to examine the phylogenetic relationship of isolates of *Pyrenopeziza* associated with light leaf spot in the UK, continental Europe, Oceania, and North America with related fungal genera and species.

| Isolate/sample code | Fungal species and lineage | Genbank accession number^a^ | | |
| --- | --- | --- | --- | --- |
|  |  | ITS rDNA | *β-tubulin* | *TEF1-α* |
| PC13 | *P. brassicae* – lineage 1 | MF187545 | MF314352 | MF314381 |
| PC17 | *P. brassicae* – lineage 1 | MF187536 | MF314353 | MF314380 |
| PC18 | *P. brassicae* – lineage 1 | MF187547 | MF314354 | MF314379 |
| PC19 | *P. brassicae* – lineage 1 | MF187546 | MF314355 | MF314378 |
| PC20 | *P. brassicae* – lineage 1 | MF187539 | MF314356 | MF314377 |
| PC22 | *P. brassicae* – lineage 1 | MF187535 | MF314357 | MF314376 |
| PC23 | *P. brassicae* – lineage 1 | MF187543 | MF314358 | MF314375 |
| PC28 | *P. brassicae* – lineage 1 | MF187538 | MF314359 | MF314374 |
| PC30 | *P. brassicae* – lineage 1 | MF187531 | MF314360 | MF314373 |
| PC32 | *P. brassicae* – lineage 1 | MF187537 | MF314361 | MF314372 |
| PC35 | *P. brassicae* – lineage 1 | MF187534 | MF314362 | MF314371 |
| PC38 | *P. brassicae* – lineage 1 | MF187544 | MF314363 | MF314370 |
| PC39 | *P. brassicae* – lineage 1 | MF187541 | MF314364 | MF314369 |
| PC45 | *P. brassicae* – lineage 1 | MF187542 | MF314365 | MF314368 |
| PC50 | *P. brassicae* – lineage 1 | MF187540 | MF314366 | MF314367 |
| 4E | *P. brassicae* – lineage 1 | MF187532 | MF314350 | MF314394 |
| 5A | *P. brassicae* – lineage 1 | MF187533 | MF314362 | MF314393 |
| FR2 (PbFr002) | *P. brassicae* – lineage 1 |  | KC342227 |  |
| CBS157.35 | *P. brassicae* – lineage 1 | MH855615 |  |  |
| IMI233716 | *P. brassicae* – lineage 1 | MF187548 | MF314351 | MF314395 |
| Cyc001 | *P. brassicae* – lineage 2 | MF143610 | MF314337 | MF314392 |
| Cyc007 | *P. brassicae* – lineage 2 | MF143611 | MF314338 | MF314391 |
| Cyc009 | *P. brassicae* – lineage 2 | MF143613 | MF314339 | MF314390 |
| Cyc011 | *P. brassicae* – lineage 2 | MF143615 | MF314340 | MF314389 |
| Cyc013A | *P. brassicae* – lineage 2 | MF143617 | MF314341 | MF314388 |
| Cyc015 | *P. brassicae* – lineage 2 | MF143619 | MF314342 | MF314387 |
| Cyc017 | *P. brassicae* – lineage 2 | MF143620 | MF314343 | MF314386 |
| Cyc023A | *P. brassicae* – lineage 2 | MF143621 | MF314344 | MN044437 |
| Cyc024A | *P. brassicae* – lineage 2 | MF143622 | MF314345 | MF314385 |
| Cyc025 | *P. brassicae* – lineage 2 | MF143623 | MF314346 | MF314384 |
| Cyc029A | *P. brassicae* – lineage 2 | MF143627 | MF314347 | MF314383 |
| Cyc031A | *P. brassicae* – lineage 2 | MK995633 | MF314349 | MF314382 |
| Genome | *Botryosphaeria dothidea* | Version 1.0^c^ | Version 1.0^c^ | Version 1.0^c^ |
| Bt4-1 | *Botrytis cinerea* |  | MG949125 |  |
| A168 | *Cadophora fastigiata* | AY249073 |  |  |
| CBS444.86 | *Cadophora finlandia* | AY249074 |  |  |
| P19 | *Cadophora gregata* | AY249070 |  |  |
| P21 | *C. gregata* | AY249071 |  |  |
| GB5129 | *Cadophora hiberna* | AF530461 |  |  |
| GB5560 | *C. hiberna* | AF530463 |  |  |
| CBS141.41 | *Cadophora luteo-olivacea* | AY249066 |  |  |
| A208 | *C. luteo-olivacea* | AY249067 |  |  |
| A174 | *C. luteo-olivacea* | AY249068 |  |  |
| A171 | *C. luteo-olivacea* | AY249069 |  |  |
| 20 | *C. luteo-olivacea* | DQ404349 |  |  |
| MM471 | *C. luteo-olivacea* | HM116747 |  |  |
| RR 87-50 | *Cadophora malorum* | AF083201 |  |  |
| A173 | *C. malorum* | AY249062 |  |  |
| A172 | *C. malorum* | AY249063 |  |  |
| A170 | *C. malorum* | AY249061 |  |  |
| A169 | *C. malorum* | AY249060 |  |  |
| A167 | *C. malorum* | AY249059 |  |  |
| A165 | *C. malorum* | AY249058 |  |  |
| A163 | *C. malorum* | AY249057 |  |  |
| A166 | *C. malorum* | AY249064 |  |  |
| A164 | *Cadophora melinii* | AY249072 |  |  |
| SHIGO-5 | *C. melinii* | AF083205 |  |  |
| Genome | *Cadophora* sp. | PRJNA243951 | PRJNA243951 | PRJNA243951 |
| REF020 | *Cadophora* sp. | JN859240 |  |  |
| C1223 | *Graphium rubrum* | AF198245 |  |  |
| C1221 | *Graphium silanum* | AY249065 |  |  |
| CBS233.39 | *Hormodendrum pyri* | MH855992 |  |  |
| Ber_02 | *Hymenoscyphus albidus* | GU586877 |  |  |
| Genome | *Hymenoscyphus fraxineus* |  | PRJEB21027 | PRJEB21027 |
| Oth_01 | *H. fraxineus* | GU586904 |  |  |
| UAMH5628 | *Leptodontidium orchidicola* | AF214578 |  |  |
| CBS412.81 | *Mollisia cinerea* | AY259135 |  |  |
| ARON3129.P | *M. cinerea* | AJ430222 |  |  |
| CBS401.78 | *Mollisia dextrinospora* | AY259134 |  |  |
| CBS401.78 (type) | *M. dextrinospora* | NR119489 |  |  |
| ARON3154.H | *Mollisia fusca* | AJ430229 |  |  |
| CBS234.71 | *M. fusca* | AY259138 |  |  |
| CBS486.48 | *M. fusca* | AY259137 |  |  |
| CBS589.84 | *Mollisia melaleuca* | AY259136 |  |  |
| ARON3139.H | *M. minutella* | AJ430223 |  |  |
| 105 | *Monilinia fructicola* |  | HQ709265 |  |
| YM09-1b | *M. fructicola* |  | HQ908770 |  |
| MLH5 | *Monilinia linhartiana* |  | LN908904 |  |
| CBS194.69 | *Neofabraea actinidiae* |  | KR859286 |  |
| CBS102871 | *Neofabraea alba* |  | KR866089 |  |
| 22-443 | *Oculimacula acuformis* |  | MN044435 | MN044438 |
| CBS 495.80 | *O. acuformis* | MH861289 |  |  |
| RAC44 | *O. acuformis* | AY266146 |  |  |
| RAE22 | *Oculimacula aestiva* | AY266145 |  |  |
| 22-433 | *Oculimacula yallundae* | AY713294 |  |  |
| CBS128.31 | *O. yallundae* | MH855154 |  |  |
| CBS282.39 | *Pezicula* sp. |  | KR859308 |  |
| A178 | *Phialophora brunnescens* | AY249079 |  |  |
| A177 | *Phialophora calyciformis* | AY249077 |  |  |
| CBS418.50 | *Phialophora cinerescens* | MH856696 |  |  |
| A176 | *Phialophora richardsiae* | AY249078 |  |  |
| CBS300.62 | *Phialocephala dimorphospora* | AY249075 |  |  |
| CBS443.86 | *Phialocephala fortinii* | AY249076 |  |  |
| CBS328.58 | *Pyrenopeziza ebuli* | MH857802 |  |  |
| CBS329.58 | *Pyrenopeziza eryngii* | MH857803 |  |  |
| CBS335.58 | *Pyrenopeziza petiolaris* | MH857804 |  |  |
| CBS336.58 | *Pyrenopeziza plicata* | MH857805 |  |  |
| ARON3150.P | *Pyrenopeziza revincta* | AJ430224 |  |  |
| CBS338.58 | *Pyrenopeziza subplicata* | MH857806 |  |  |
| CNF:2/10097 | *Pyrenopeziza velebitica* | NR158942 |  |  |
| CNF 2/10097 (type) | *P. velebitica* | MF593628 |  |  |
| Genome | *Rhynchosporium commune* |  | PRJEB12897 |  |
| H25 (Haplotype 25) | *R. commune* | HM627492 |  |  |
| 27DG09 | *Rhynchosporium orthosporum* |  | MN044436 | MN044439 |
| H4 (Haplotype 4) | *R. orthosporum* | HM627471 |  |  |
| TZ25 | *Sclerotinia sclerotiorum* |  | AY312374 |  |
| ARON3188.H | *Tapesia cinerella* | AJ430228 |  |  |

1. ITS rDNA = internal transcribed spacer (ITS) region of ribosomal DNA (rDNA); *β-tubulin* = *beta-tubulin* gene; *TEF1-α* = *translation elongation factor 1-α* gene. Only a partial ITS1 rDNA sequence could be amplified from the type herbarium specimen of *P. brassicae* (IMI81823), and was deposited in GenBank as Accession MN028386. Underlined sequences were used for the concatenated analyses of all three loci.
2. *Botryosphaeria dothidea* genome available at <https://genome.jgi.doe.gov/Botdo1_1/Botdo1_1.home.html> (accessed 21 May 2019).
